# Spatiotemporal Distribution of Household WEEE and Anthropogenic Mineral Reserves in China from 1978 to 2050

**DOI:** 10.1021/acs.est.6c02246

**Published:** 2026-03-25

**Authors:** Zongqi Yu, Yifan Gu, Chenyang Shuai, Xi Chen, Ming Xu, Bu Zhao

**Affiliations:** † Tepper School of Business, 33850 Carnegie Mellon University, Pittsburgh, Pennsylvania 15213, United States; ‡ Institute of Circular Economy, College of Materials Science and Engineering, 529069 Beijing University of Technology, Beijing 100124, P.R. China; § School of Management Science and Real Estate, 1259 Chongqing University, Chongqing 400044, P.R. China; ∥ College of Economics and Management, 26463 Southwest University, Chongqing 400715, P.R. China; ⊥ School of Environment, 12442 Tsinghua University, Beijing 100084, P.R. China; # Department of Environmental and Sustainable Engineering, University at Albany, State University of New York, Albany, New York 12222, United States

**Keywords:** WEEE, material flow analysis, anthropogenic mineral, household appliance, resource recovery

## Abstract

China, the world’s largest generator of waste electrical and electronic equipment (WEEE), faces growing challenges in managing household WEEE and recovering embedded resources. This study develops a long-term, provincial-level assessment of household WEEE generation and anthropogenic mineral (AM) reserves in China from 1978 to 2050. Using a stock-based material flow analysis, we quantify in-use stocks, WEEE generation, and associated AM for 10 household appliance categories (14 types) across 31 provinces. Annual household WEEE generation exceeded 400 million units in 2023, dominated by cell phones, computers, air conditioners, and washing machines, with eastern and southeastern provinces contributing the largest volumes. Embedded AM reserves in 2023 include about 228 thousand tonnes of copper (9.4% of national recycled copper), 21 tonnes of gold (5.6% of primary production), and 7 tonnes of palladium (50% of primary production), with a theoretical metal value of over 5 billion USD. Yet, existing formal recycling infrastructure captures only ∼30% of this potential, owing to pronounced spatial and category-specific mismatches. Our results identify critical gaps and province-level hotspots, providing a quantitative basis for spatially targeted WEEE management and infrastructure planning to enhance resource recovery in China’s transition toward a circular economy.

## Graphical abstract

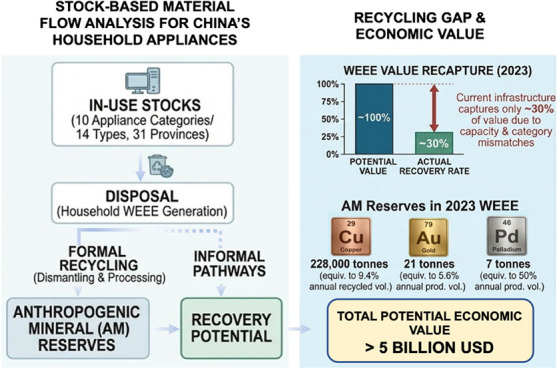

## 1 Introduction

Waste Electronic and Electrical Equipments (WEEEs), commonly known as e-wastes, refer to discarded electrical and electronic devices that operate using electrical currents or electromagnetic fields, such as televisions, washing machines, computers, and cell phones that are no longer in use or have reached the end of their service life cycle.^1^ Driven by rapid technological innovations, improved affordability, and shorter product life expectancy, global WEEE generation has risen sharply in recent decades.^2,3^ As evidenced in a relevant study, WEEE is the fastest-growing waste stream worldwide, with annual generation increasing by about 3–5% and total volumes reaching approximately 62 million tonnes in 2022.^4–6^ At the same time, WEEE contains substantial quantities of valuable metals and other materials, making it an important source of anthropogenic minerals (AM) and a key lever for resource recovery and circular economy strategies.^7^ Taking cell phones as an example, one metric ton of waste cell phones can contain up to 280g of gold and 2000g of silver, which are roughly 60 and 13 times the content level found in raw ores and the potential metal value can be up to 23,000 USD per tonne.^8^ Therefore, appropriate WEEE recycling can not only deliver considerable economic benefits, but also reduce secondary waste generation, prevent the release of hazardous contents, and avoid energy and emissions associated with primary production.^9–11^

Household appliances are a major subgroup of the EEEs that are primarily used in residential settings for household functions, including large appliances such as washing machines and refrigerators, small appliances such as microwaves, and consumer electronics such as cameras and cell phones. These EEEs contain significant quantities of critical raw materials such as steel, copper, aluminum, and gold. However, unlike the WEEEs from commercial or industrial settings, household WEEEs are typically generated in highly dispersed locations and often lack centralized collection channels. In many regions, retired household appliances are collected informally by itinerant peddlers or discarded with mixed municipal waste, resulting in low collection rates, uncontrolled treatment, and the loss of critical secondary resources.

In response to these challenges, over the past two decades, many economies have introduced dedicated WEEE management frameworks. In the European Union, the WEEE Directive (2012/19/EU) has transitioned toward more stringent targets, requiring a minimum collection rate of 65% of the average weight of EEEs placed on the market in the three preceding years.^5^ Similarly, Japan has established a unique dual-track legislative system: the Home Appliance Recycling Law (HARL) specifically targets large appliances (televisions, air conditioners, washing machines, and refrigerators), mandating a sophisticated “front-end” collection system where retailers and manufacturers share the responsibility for logistics and high-purity material recovery.^12^ Beyond these regions, countries like South Korea and the United States have increasingly adopted Extended Producer Responsibility (EPR) programs, with a recent shift toward “Right to Repair” legislation aimed at extending the lifespan of household electronics before they enter the waste stream.^13^ Furthermore, recent research trends highlight a transition from simple volume-based recycling to value-based recovery through “urban mining”, focusing on high-value and critical metals in WEEEs and treating both in-use and discarded devices as secondary resource deposits within a circular economy framework.^14^

China is now one of the largest consumers of EEEs and the largest producer of WEEEs in the world.^7^ Since the early 2000s, rapid income growth, urbanization, and technological upgrading have made household appliances indispensable in Chinese homes, leading to a surge in both in-use stocks and end-of-life wastes. In response to this situation, the Chinese government has introduced a range of policies to promote formal recycling and resource recovery. A key step was the formal introduction of the AM concept in 2010, which explicitly designates the valuable fractions of WEEEs and other secondary resources, such as iron, nonferrous and precious metals, plastic, and rubber, as recoverable mineral reserves.^7,15^ Building on this conceptual framework, the scope of regulated categories of WEEEs was expanded from 5 to 14 by 2015.^16^ To further promote the maximum resource recovery of WEEEs through legal channels, China introduced the “Administrative Measures for Eligibility License for Disposal of Waste and Discarded Electrical and Electronic Products” to distribute licenses for qualified dismantling companies, which marks the nation’s ongoing efforts to enhance sustainable recycling practices and resource recovery from WEEEs.^17^

To optimize resource recovery, mitigate environmental impacts, and inform evidence-based policy, a growing body of research has examined WEEE generation, stocks, and recycling in major economies and provided important methodological and empirical insights (Table 1). For Europe, Parajuly et al. (2017) conducted a dynamic material flow analysis (MFA) for 61 categories of household EEEs in Denmark, projecting that WEEE generation will reach 134 thousand tonnes (approximately 23 kg/capita) by 2025. Their study highlights a significant resource recovery gap, noting that despite a 50% formal collection rate, nearly 10% of small appliances are lost to residual waste while substantial stocks remain “hibernating” in households.^18^ Similar stock-driven MFA studies for specific product groups, such as laptops in Greece and discarded refrigerators in several EU member states, have revealed how changing lifetimes, market saturation, and policy targets affect future WEEE generation and embedded critical metals.^21,22^ For U.S., Althaf et al. (2021) conducted a dynamic, high-resolution MFA of 20 major consumer electronics in U.S. households from 1980 to 2018, revealing that total e-waste mass has declined by 10% since 2015 due to the phase-out of heavy CRT displays, while its composition has shifted toward critical materials like cobalt and away from regulated hazardous substances.^13^ In Australia, dynamic MFA has been used to forecast EEEs put-on-market, in-use stocks and e-waste flows under different saturation and growth scenarios, highlighting the sensitivity of long-term projections to assumptions about market maturity.^20^ Collectively, these studies demonstrate that transparent, stock-driven MFA is a powerful framework for characterizing long-term WEEE and secondary resource dynamics and for informing collection, recycling, and infrastructure planning.

**Table 1:** Comparison of the Scope of This Study with Previous Studies

Time Period	Spatial Coverage	WEEE Scope	Categories	References
1980–2018	National (U.S.)	Household Appliances	20	^13^
1990–2025	National (Denmark)	Household Appliances	61	^18^
1971–2030	National (Canada)	Household and Commercial Appliances	51	^19^
2010–2030	National (Australia)	Household Appliances	16	^20^
1983–2040	National (Greece)	Laptop	1	^21^
1935–2050	National (Colombia)	Refrigerator	1	^22^
1989–2004	National (China)	Household Appliances	5	^23^
2003–2007	National (China)	Household Appliances	5	^24^
2003–2006	Regional (Beijing)	Household Appliances	5	^25^
2001–2010	National (China)	Household Appliances	5	^26^
2010–2012	National (China)	Household Appliances	5	^27^
2010–2015	National (China)	Household and Commercial Appliances	14	^28^
2000–2015	Regional (Beijing)	Household Appliances	5	^29^
2010–2020	National (China)	Household and Commercial Appliances	15	^7^
1990–2035	National (China)	Household Appliances	1	^30^
1978–2050	National (China)	Household Appliances	10	This study

Building on this global experience, from 2015 onward, a series of studies have analyzed WEEEs in China using stock-driven MFA or related approaches, with a strong emphasis on quantifying the volume of AM embedded in WEEEs. For example, Kong and Liu (2018) characterized the material metabolism of urban household durable goods across 31 provinces, estimating a total provincial stock of 131 million tonnes in 2014 and revealing a strong correlation between material accumulation and regional GDP.^31^ Gu et al. (2016) investigated the growth of WEEE generation in Beijing and examined whether the recycling of WEEEs can meet the resource demands of the electronics industry.^29^ Complementing this, Zeng et al. (2016) specifically identified the untapped recycling potential of “new” WEEE categories (such as printers and monitors) beyond the initial mandate, predicting their increasing significance in future waste streams.^28^ As a follow-up study, Zeng et al. (2020) further measured the potential of recycled metals from a broader range of WEEEs, projecting that their economic value could reach 400 billion USD by 2050 and meet the entire projected consumption of 20 key materials.^7^ In addition to metal, existing studies also discuss the recycling of plastics from WEEEs. Sun et al. (2022) quantified the stocks and flows of plastics contained in five categories of households in China over 1978–2016 and mapped their province-specific distribution through a dynamic stock-driven MFA model.^32^ Complementing these MFA studies, life-cycle assessment combined with machine learning has also been employed to evaluate the environmental impacts and mitigation potential of WEEE plastics recycling, thereby linking circular-economy strategies with China’s carbon-neutrality targets.^33^

Despite these advances, important knowledge gaps remain. First, most existing studies provide only national or selective provincial estimates over short timeframes, typically over one to two decades, and only for five “regulated” household appliance categories. They therefore fail to capture the full spatial and long-term evolution of WEEE and AM accumulation across China. Second, methodological challenges, such as separating waste from dormant stocks and sparse historical product-specific material composition data, limit a clear understanding of the long-term, province-specific evolution of AM reserves in WEEE. Third and most importantly, most studies only focus on the theoretical amounts of AM embedded in WEEEs, the actual recovery rate achieved through formal channels, and consequently, the mismatch between actual WEEE generation and existing recycling capacity, remains largely unquantified. These gaps, in turn, hamper efforts to assess the environmental implications of current WEEE management practices and to design targeted interventions to reduce uncontrolled treatment and associated emissions.

To address these gaps, this study provides a long-term, provincial-level assessment of household WEEE generation and AM reserve in China from 1978 to 2050. Specifically, we apply a stock-based MFA (SMFA) approach combined with Weibull lifetime modeling to reconstruct the evolution of annual in-use stocks, WEEE generation, and associated AM recovery potential for 10 categories (14 types) of household appliances in 31 provinces over both historical (1978–2023) and projected (2024–2050) periods. We then combine the modeled WEEE and AM flows with firm-level and provincial statistics for 109 certified dismantling enterprises to estimate licensed and actual recycling volumes, and to derive, for each province and metal, the amount of AM recovered by formal facilities and the corresponding unrecovered remainder that is likely subject to inappropriate or informal treatment. Our results show that annual household WEEE generation exceeded 400 million units in 2023, with cell phones, computers, air conditioners, and washing machines as the largest contributors. Geospatial analysis reveals that eastern, southeastern, and southwestern provinces dominate the WEEE generation, where economic development may accelerate replacement cycles. The AM reserves embedded in household WEEE in 2023 include about 228 thousand tonnes of copper (equivalent to 9.4% of national copper recycling), 21 tonnes of gold (5.6% of annual production), and 7 tonnes of palladium (50% of annual production), corresponding to a theoretical economic potential of over 5 billion USD. Yet current formal recycling infrastructure captures only about 30% of this potential value at the national level, with pronounced province-level mismatches between WEEE/AM generation and licensed recycling capacities.

The main contributions and novelty of this paper are as follows:

- We establish the first long-term (1978–2050), province-resolved SMFA of household WEEE and embedded AM reserves in China, covering 10 categories and 14 types of household appliances. By jointly reconstructing historical dynamics (1978–2023) and projecting future trends (2024–2050), the study extends previous national-scale assessments that typically focused on shorter time horizons, fewer categories, or aggregated spatial units.
- We explicitly characterize the spatiotemporal dynamics of household WEEE and AM accumulation by reconstructing provincial trajectories of in-use stocks, WEEE generation, and AM reserves over more than seven decades, and by revealing distinct provincial “pathways” of stock saturation and obsolescence as well as shifts in the spatial concentration of key metals such as copper and precious metals.
- We are among the first to explicitly quantify and map the pronounced province-level mismatches between WEEE/AM generation and licensed recycling capacities in China. By combining SMFA outputs with firm-level and provincial-level data on 109 certified dismantling enterprises, their licensed capacities, and actual treatment volumes, we derive province- and metal-specific capture rates and explicitly quantify the amounts of AM that are recovered by formal facilities versus the unrecovered remainder that is likely subject to inappropriate or informal treatment.
- Building on these identifications of spatial mismatches, we provide region-specific implications for optimizing WEEE management in China, including the prioritization of provinces with high current or future AM reserves but insufficient formal capacity, and differentiated recommendations for national and provincial policymakers, recycling enterprises, and technology deployment. These insights offer a quantitative basis for spatially targeted infrastructure planning and policy design to support China’s transition toward a circular economy.

Subsequent sections are organized as follows. Section 2 introduces the methodologies adopted in the four stages of our study. Section 3 presents the main results and insights. Lastly, Section 4 discusses implications for the effectiveness of the existing WEEE management system and provides region-specific recommendations for policymakers and other stakeholders.

## 2 Methods

### 2.1 Scope and Data

This study focuses on the WEEE generation and material recovery potential from household appliances in mainland China. The 10 categories of household appliances (14 types in total) included in this paper are air conditioners (AC), range hoods (VT), refrigerators (FR), washing machines (WM), water heaters (WH), microwave ovens (MW), televisions (TV), cameras (CAM), computers (CP), and cell phones (PHONE). To account for significant technological and composition heterogeneity within each household appliance category, we further classify products into subtypes based on their core technologies. Televisions are further categorized into subtypes of Liquid Crystal Display (TV-LCD) and Cathode Ray Tube (TV-CRT) televisions. Computers are further categorized into LCD desktops (CP-Desk-LCD), CRT desktops (CP-Desk-CRT), and laptops (CP-Lap). Water heaters are further categorized into electric (WH-EWH) and gas (WH-GWH) water heaters.

To ensure a detailed and internally consistent assessment, we compiled multisource data from peer-reviewed papers, governmental statistics, industry reports, and news including:

- Statistics of population and household size in urban and rural areas by region from 1978 to 2023;^34,35^

- Statistics of main durable goods owned per 100 households, in urban and rural areas, by region from 1978 to 2023;^35^
- Statistics of estimated WEEE imports from 2010 to 2023;^28,36–39^

- Statistics of the licensed capacity of government-certified recycling companies in 2024;^40^
- Statistics of the capacity of government stipend-supported recycled WEEEs in 2013;^41,42^

- Statistics of the capacity breakdown of recycled WEEEs from 2011 to 2023;^40–46^

- Statistics of the average weights of main durable goods from 1995 to 2023;^7,47–50^

- Statistics of the mineral composition of main durable goods in 1983, 1993, 2004, 2006, 2011, and 2015;^7,31,51–55^

- Statistics of WEEE subtypes shares of televisions, computers, and water heaters;^7,28^

- Weibull parameters of the lifetime of durable goods;^7,47,56^

- Metal prices from the past 5 years.^57^

The generation and recycling of WEEEs are influenced by exogenous factors such as regulations, policies, and technological changes.^58^ However, these drivers are hard to quantify consistently over long time horizons.^7^ Therefore, this study mainly focuses on the endogenous variables to estimate the generation and recycling potential of 14 types of WEEE in all 31 provinces in mainland China from 1978 to 2050 through the SMFA framework. The framework consists of four main stages (Figure 1), i.e., in-use stock estimation, WEEE generation estimation, AM potential quantification, and recycling gap estimation, followed by a sensitivity analysis of key parameters. More detailed data information can be found in the (Supporting Information SI) and the missing data are estimated using linear interpolation.

**Figure 1:**
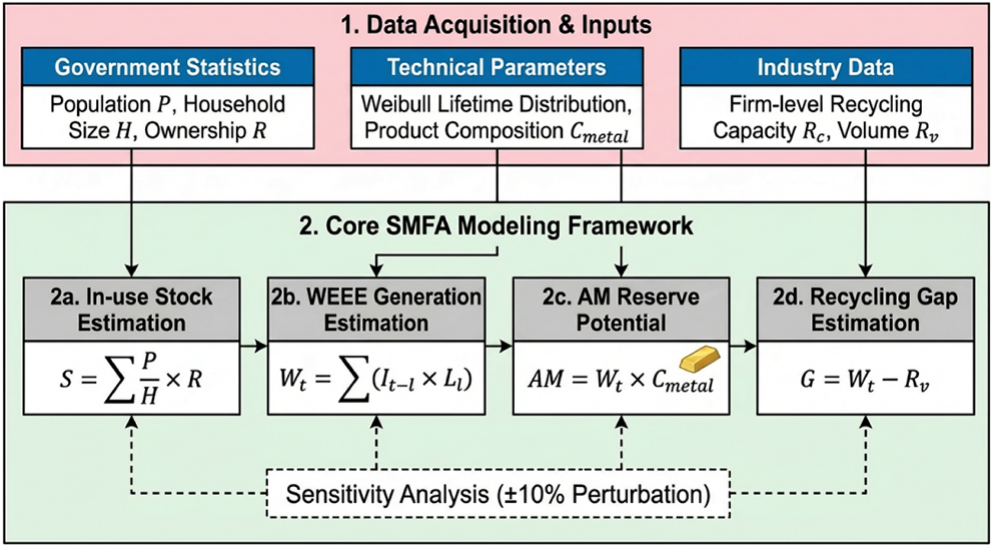
Integrated stock-based MFA framework.

### 2.2 In-Use Stock Estimation

The in-use stock estimation aims to quantify the number of household appliances that are actively being used in each province annually. The in-use stock of household appliances *i* in province *j* and year *t* is calculated as

1 $$S_{ijt}=p_{jt}^{U}÷h_{jt}\times r_{ijt}^{U}÷100+p_{jt}^{R}÷h_{jt}\times r_{ijt}^{R}÷100$$

Where *S*_*ijt*_ denotes the in-use stock (units) of type *i* appliance in province *j* in year *t*. $p_{jt}^{U}$ and $p_{jt}^{R}$ represents the urban and rural population in province *j* in year *t*. *h_jt_* is the average household size of province *j* in year *t*. $r_{ijt}^{U}$ and $r_{ijt}^{R}$ represent the ownership of type *i* durable good per 100 households for urban and rural population in province *j* in year *t*.

### 2.3 WEEE and AM Generation Estimation

Various methods have been developed to estimate WEEE generation, but most of them rely on sales and purchase data, such as the direct MFA, the Market Supply Method, the Carnegie Mellon Method, and the Consumption and Use Method. However, imports, overseas purchases, secondary markets, and refurbished devices create substantial uncertainty in sales statistics, especially when spatially disaggregated estimates are required. To avoid these limitations, we adopt an SMFA method that infers outflows from in-use stock dynamics and lifetime distributions, rather than relying on sales data to estimate WEEE generation for the historical period 1978–2023. The lifetime of each appliance type is represented by a Weibull distribution, with parameters taken from established literature to ensure methodological continuity and comparability with prior studies. The basic stock balance is expressed as

2 $$S_{ijt}=I_{ijt}-O_{ijt}+S_{ij\left(t-1\right)}$$

Where *S*_*ijt*_ denotes the in-use stock (units) of type *i* appliance in province *j* in year *t*. *I*_*ijt*_ and *O*_*ijt*_ denote the units of inflow to and outflow from the in-use stock of type *i* durable goods in province *j* in year *t*. To be more specific, the inflow refers to the appliances entering active use (e.g., through purchases of new devices, or resales and reuse of dormant devices) while outflow corresponds to appliances leaving active use due to failure, retirement, or becoming dormant (i.e., the WEEE generation). In the framework of SMFA, outflows can be derived implicitly from the in-use stock changes and the lifetime distribution of appliances shown below:

3 $$O_{ijt}=\overset{t}{\underset{l=1}{\sum }}I_{ij\left(t-l\right)}\times L_{i}\left(t-l,\mu_{i},\sigma_{i}\right)$$

Where *I*_*ij*(*t*–l)_ represents the inflow of type *i* durable goods in province *j* in year *t*–*l*, and *l* is the number of years since entry into use. *L*_*i*_(*t*–*l*,*μ*_*i*_,σ_i_) is the retirement rate of type *i* durable goods that entered use *l* years earlier. Specifically, the retirement rate *L*_*i*_ is modeled using a Weibull distribution, which is a widely used and empirically validated approach for representing the lifetime and failure behavior of machinery and household appliances.^28,59^ For appliance type *i*, the probability density function is

4 $$L_{i}\left(t-l,\mu_{i},\sigma_{i}\right)=\{\begin{array}{l} \frac{\sigma_{i}}{\mu_{i}}\times {\left(\frac{t-l}{\mu_{i}}\right)}^{\sigma_{i}-1}\times e^{-{\left(\frac{t-i}{\mu_{i}}\right)}^{\sigma_{i}}},t-l\geq 0\\ 0,t-l<0 \end{array}$$

Where *μ*_*i*_ and σ_i_ are the scale and shape parameters of the Weibull distribution, respectively (Table S2). Figure 2 illustrates the Weibull lifetime assumptions for the 14 appliance types. Panel (a) displays the cumulative retirement functions, which separate the remaining in-use stock (above the curve) from the cumulative obsolete fraction (below the curve) at each age. Panel (b) shows the corresponding annual retirement rates, which are used to determine the yearly WEEE outflows and associated AM releases for each product type. However, recent survey-based work has shown that service lifetimes of household appliances can vary substantially across regions due to differences in income levels, consumption habits, and cross-regional flows of second-hand products through informal networks.^60^ Due to the lack of spatially and product-resolved lifetime profiles that cover all 14 appliance types and 31 provinces, we adopt nationally calibrated Weibull parameters as our baseline for all provinces. We then assess the implications of regional lifetime variation through a dedicated macro-regional sensitivity analysis described in Section 2.5.

**Figure 2:**
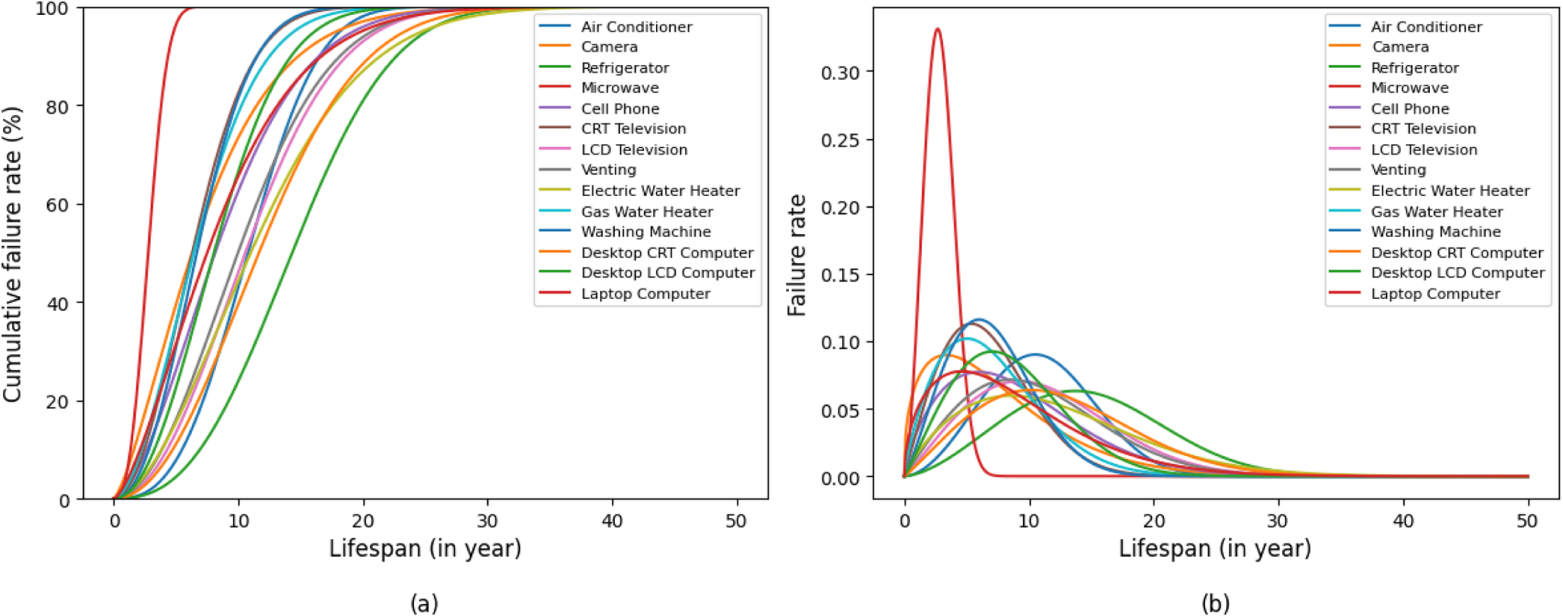
Weibull lifetime distributions for household appliances: (a) cumulative retirement (failure) functions; (b) annual retirement (failure) rate functions for the 14 product types.

In addition to reconstructing historical WEEE generation, we project future WEEE generation by product category to 2050 using a lifecycle-consistent modeling framework that distinguishes between technologies still in the growth phase and those that have already peaked and are in decline. For product groups that continue to exhibit monotonic growth over the historical period, we apply a logistic growth function to capture the characteristic S-shaped diffusion of emerging technologies:

5 $$G_{i}\left(t\right)=\frac{K_{i}}{1+\exp\left[-a_{i}\left(t-b_{i}\right)\right]}$$

Where *G*_*i*_(*t*) is the annual WEEE generation of product type *i* in year *t*; *K*_*i*_ is the saturation level (asymptotic maximum generation); *a*_*i*_ is the intrinsic growth rate; and *b*_*i*_ is the inflection point (the time at which *G*_*i*_(*t*) reaches half of *K*_*i*_). For categories such as CRT and LCD televisions, which display a clear rise-peak-decline trajectory due to technological substitution, we employ a Hubbert-type (Gaussian bell-shaped) function to represent both the historical adoption phase and the subsequent phase-out:

6 $$H_{i}\left(t\right)=A_{i}\exp\left[-\frac{{\left(t-t_{0,i}\right)}^{2}}{2\sigma_{H,i}^{2}}\right]$$

Where *H*_*i*_(*t*) is the annual WEEE generation of product type *i* in year *t*; *A*_*i*_ is the peak generation level; *t*_0,*i*_ is the year of peak generation; and σ_*H*,*i*_ controls the spread of the rise-peak-decline profile over time. For each of the 14 product types, the model parameters (*K*_*i*_,*a*_*i*_,*b*_*i*_) or (*A*_*i*_,*t*_0_,_*i*_,σ_*H*,i_) are estimated by nonlinear least-squares fitting to the modeled historical WEEE generation series over 1978–2023. The calibrated functions are then extrapolated to 2050 to obtain category-specific WEEE generation forecasts.

Then, the AM reserve embedded in WEEE is further estimated based on the modeled WEEE generation. In this study, we consider three groups of materials: (i) base metals and associated nonmetallic materials (Cu, Fe, Al, Steel, Plastic, Glass), (ii) economically valuable metals (Ba, Cd, Cr, Sb, Pb, Sn, Zn), and (iii) precious metals (Au, Ag, Co, In, Pd, Ru), following previous studies.^7,28,52^ Specifically, the total amount of metal *k* contained in WEEE generated in province *j* and year *t* is

7 $$m_{jkt}=\underset{i}{\sum }w_{it}\times O_{ijt}\times c_{ikt}$$

### 2.4 Recycling Gap Estimation

During the recycling process, household WEEEs may be handled by a variety of actors, such as informal peddlers for large household appliances, gray markets for cell phone and camera parts, and formal, government-certified recycling companies.^61^ In the scope of this study, we mainly focus on the 109 licensed dismantling companies certified by the Ministry of Ecology and Environment, for which consistent data on licensed treatment capacity and actual recycling volumes are available. These companies are officially authorized to treat only the five regulated e-waste categories, i.e., air conditioners, computers, refrigerators, washing machines, and televisions. The national-level actual recycling volumes and licensed recycling capacities for these companies are collected from the Industrial White Papers.^44^ Based on these data, we distinguish two empirical scenarios for 2013–2023: an “actual-operation” scenario, using the reported annual volumes of WEEE treated by certified facilities, and a “licensed-capacity” scenario, using the designed treatment capacities of the 109 enterprises. These two scenarios are later compared with the modeled AM reserves to quantify, for each metal and year, the gap between theoretical availability, potential recovery under full utilization, and realized recovery. Using these data, together with firm-level data on government subsidy claims for 2013, and provincial distributions of recycled WEEE reported in official documents, we derive the shares of each WEEE type in total recycled volume and the shares of each province in national recycling.^40–42,44^ Figure S2 provides more detailed information on the method for deriving the breakdowns. The final licensed and actual recycling volumes of the WEEEs at the provincial level are determined by

8 $$\begin{array}{l} \overset{®}{R_{ijt}\;}=\min\left(\overset{®}{Q_{ijt}\;},O_{ijt}\right)\\ \underset{\_}{R_{ijt}\;}=\min\left(\underset{\_}{Q_{ijt}\;},O_{ijt}\right) \end{array}$$

Where $\overset{®}{R_{ijt}\;}$ and *R*_*ijt*_ denote the number of units of types *i* WEEE recycled in province *j* and year *t* under the licensed capacity and actual recycling volume. $\overset{®}{Q_{ijt}\;}$ and *Q*_*ijt*_ are the corresponding licensed-capacity and actual recycling volume (in units) of type *i* WEEE in province *j* during year *t*. The provincial and type-specific capacities are obtained by disaggregating the national licensed capacity and actual recycling volume, $\overset{®}{Q_{t}\;}$ and *Q*_*t*_ , using the shares of each WEEE type and province in total recycling:

9 $$\begin{array}{l} \overset{®}{Q_{ijt}\;}=\overset{®}{Q_{t}\;}\times \Delta i\times \Delta j\\ \underset{\_}{Q_{ijt}\;}=\underset{\_}{Q_{t}\;}\times \Delta i\times \Delta j \end{array}$$

Where Δ*i* denotes the share of type *i* in total recycled WEEEs, Δ*j* denotes the share of province *j* in national recycled WEEEs. WEEEs that are not treated by certified facilities are assumed to be disposed of together with municipal solid waste or processed through informal channels. The corresponding amounts of unrecycled WEEEs are

10 $$\begin{array}{l} \overset{®}{W_{ijt}\;}=O_{ijt}-\overset{®}{R_{ijt}\;}\\ \underset{\_}{W_{ijt}\;}=O_{ijt}-\underset{\_}{R_{ijt}\;} \end{array}$$

Where $\overset{®}{W_{ijt}\;}$ and *W*_*ijt*_ denote the units of types *i* WEEE in province *j* in year *t* that are not recycled under the licensed-capacity and actual-operation scenarios, respectively. It should be emphasized that, due to data limitations, the flows treated by informal collectors, dismantlers, and refurbishment networks are not explicitly modeled in this study. Consequently, the “unrecycled” WEEE quantified in eqs 8–10 should be interpreted as WEEE not treated by certified facilities, rather than as flows for which no material recovery occurs at all. In reality, a substantial share of these “unrecycled” units, especially large household appliances and information and communication devices, may be partially processed by informal actors, often with lower material recovery efficiency and weaker environmental controls. Therefore, the recycling gaps reported in this paper represent conservative upper bounds on the shortfall between theoretical AM reserves and formal recovery.

Regarding the material recovery potential, the amounts of material *k* that are potentially recoverable under the licensed-capacity and actual-operation scenarios are

11 $$\begin{array}{l} \overset{®}{m_{jkt}\;}=\underset{i}{\sum }w_{it}\times \overset{®}{R_{ijt}\;}\times c_{ikt}\\ \underset{\_}{m_{jkt}\;}=\underset{i}{\sum }w_{it}\times \underset{\_}{R_{ijt}\;}\times c_{ikt} \end{array}$$

Where *m*_*jkt*_ denotes the amount of material *k* available in WEEE in province *j* and year *t*. $\overset{®}{m_{jkt}\;}$ and *m*_*jkt*_ denote the corresponding amount of material *k* in province *j* and year *t* under the licensed-capacity and actual-operation scenarios. *w*_*it*_ is the average weight of type *i* appliance in year *t*. *c*_*ikt*_ is the mass fraction of material *k* in type *i* appliance in year *t*.

To estimate the economic value of material recovery under the licensed-capacity and actual-operation scenarios, we use the arithmetic mean of the 5-year historical maximum and minimum nominal prices for tradable materials (Cu, Fe, steel, Al, Pb, Sn, Zn, Au, Ag, Co, In, Pd) for value estimation. All prices are expressed in current (nominal) USD without adjustment for inflation:

$$\begin{array}{l} V_{t}=\underset{j}{\sum }\underset{k}{\sum }m_{jkt}\times v_{k}\\ \overset{®}{V_{t}\;}=\underset{j}{\sum }\underset{k}{\sum }\overset{®}{m_{jkt}\;}\times v_{k}\\ \underset{\_}{V_{t}\;}=\underset{j}{\sum }\underset{k}{\sum }\underset{\_}{m_{jkt}\;}\times v_{k} \end{array}$$ 12

Where *V*_*t*_, $\overset{®}{V_{t}\;}$ and *V*_*t*_ denote the theoretical maximum nominal value of recoverable material contained in WEEEs, and the values realized under the licensed-capacity and actual-operation scenarios in year *t*. *v*_*k*_ is the average of the 5-year highest and lowest market price of the material *k*. The resulting values represent the theoretical gross value of metals contained in WEEE and recoverable under the two scenarios, respectively. They do not account for costs associated with collection, transportation, dismantling, processing losses, environmental compliance or profit margins, and therefore should not be interpreted as net economic benefits for recycling enterprises.

### 2.5 Sensitivity Analysis

To assess the robustness of our results, we conducted two sensitivity analyses focusing on the Weibull distribution parameters, which critically influence lifespan modeling and subsequent WEEE generation estimates. Specifically, we evaluated how variations in the scale (*μ*) and shape (σ) parameters affect the projected WEEE volumes by applying a ±10% perturbation to each parameter independently and quantifying the resulting deviations in generation estimates.

In addition to the product-level parameter perturbations, we conducted a regional sensitivity analysis to explore how spatial variation in service lifetimes could influence our WEEE projections. Following the macro-regional classification of the National Bureau of Statistics, the 31 provinces are grouped into four regions: eastern, central, western and northeastern China. Empirical evidence from recycling-plant surveys indicates that effective lifetimes of major household appliances tend to be shorter in economically more developed eastern regions and longer in less developed western and northeastern regions.^60^ To reflect this pattern in a stylized way, we adjust the Weibull scale parameter *μ*_*i*_ for selected appliance types by region, while keeping the shape parameter σ*_i_* constant. Specifically, in the regional-lifetime scenario we decrease *μ*_*i*_ by 10% for eastern provinces, leave *μ*_*i*_ unchanged for central provinces, and increase *μ*_*i*_ by 10% for western and northeastern provinces. Because detailed, region-specific lifetime information is currently available only for the five large regulated appliances, we implement this regional sensitivity analysis as a case study for two representative product types: refrigerators and washing machines. For these two categories, we rerun the SMFA for the period 1978–2050 under the region-differentiated set of *μ*_*i*_ values and compare the resulting WEEE generation trajectories with those obtained under the baseline, nationally uniform lifetime parameters.

## 3 Results

This section presents the results based on household WEEE generation estimations from 1978 to 2050. In addition, WEEE importation data from 1978 to 2023 is provided as a reference to the readers but does not contribute to the recycling and material recovery estimation due to the lack of detailed composition on WEEE types. If not specified otherwise, this paper keeps the unit for WEEE in Section 3.1, 3.2, and 3.3 as “unit” except for the imported ones (as all governmental statistics on WEEE recycling capacity are based on “unit” but not weight), and Section 3.4 measures the material recovery using the unit of metric tonne.

### 3.1 National In-Use Stock of Household Appliances

Driven by rapid economic development and rising living standards, the in-use stock of almost all household appliance types in China increased sharply between 1990 and 2010 (Figure 3). This growth primarily reflects the combined effects of GDP expansion, urbanization, and improvements in household income over this period. Large household appliances such as air conditioners, refrigerators, microwave ovens, range hoods, and water heaters exhibit relatively steady stock growth, consistent with their role as “necessity” goods with basic and stable functions. By 2023, the in-use stock of air conditioners exceeded 760 million units, computers exceeded 230 million units, televisions were around 548 million units, and refrigerators in use just exceeded 520 million units.

**Figure 3:**
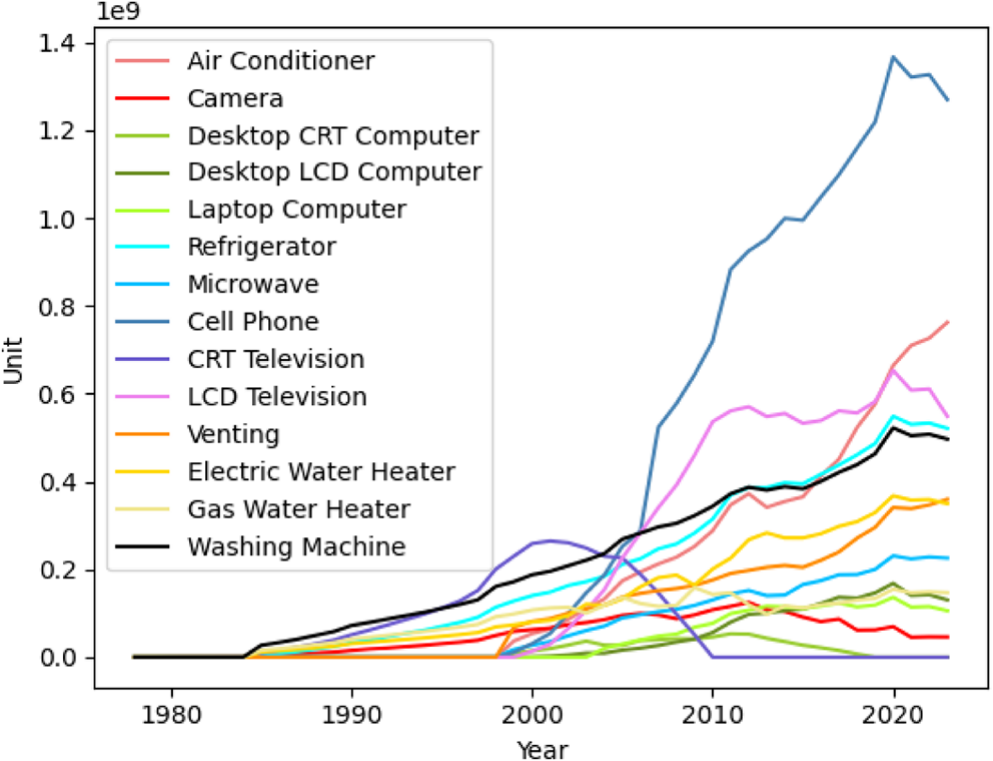
National-level in-use stock of household appliances by type from 1978 to 2023.

In contrast, cell phones experienced an almost exponential growth in in-use stock from the early 2000s to around 2020, when the total number of phones in use peaked at approximately 1.36 billion units. This reflects both rapid diffusion and shortened replacement cycles associated with smartphone adoption. In 2020–2021, noticeable fluctuations in demand and in-use stock can be observed across several categories, which are largely attributed to the COVID-19 pandemic. Supply chain disruptions, changes in work-from-home and entertainment patterns, and heightened economic uncertainties jointly altered purchasing behavior, temporarily slowing growth for some products while accelerating upgrades for others.

Table S1 provides a further breakdown of the in-use stock by technology type (e.g., LCD and CRT televisions). Notably, some subcategories, such as CRT televisions and cameras, have declined substantially in recent years due to technological obsolescence. Taken together, these stock trajectories suggest that future WEEE generation from basic large appliances will increasingly be driven by replacement in a saturated market, whereas information and communication technologies with faster turnover (e.g., cell phones, computers) will generate earlier and more concentrated waves of end-of-life products. The rapid phase-out of legacy technologies also indicates that, although their stocks are shrinking, they still contribute specific materials such as leaded glass and certain legacy components to the waste stream.

### 3.2 WEEE Generation Estimation

Based on the in-use stock estimates, we further derive annual WEEE generation using the SMFA framework for all 14 product types in each province. Specifically, we first analyze historical WEEE generation over 1978–2023 and then discuss projections up to 2050. The resulting total and spatial-temporal resolved WEEE generation patterns are demonstrated in Figures 4 and 5, respectively.

**Figure 4:**
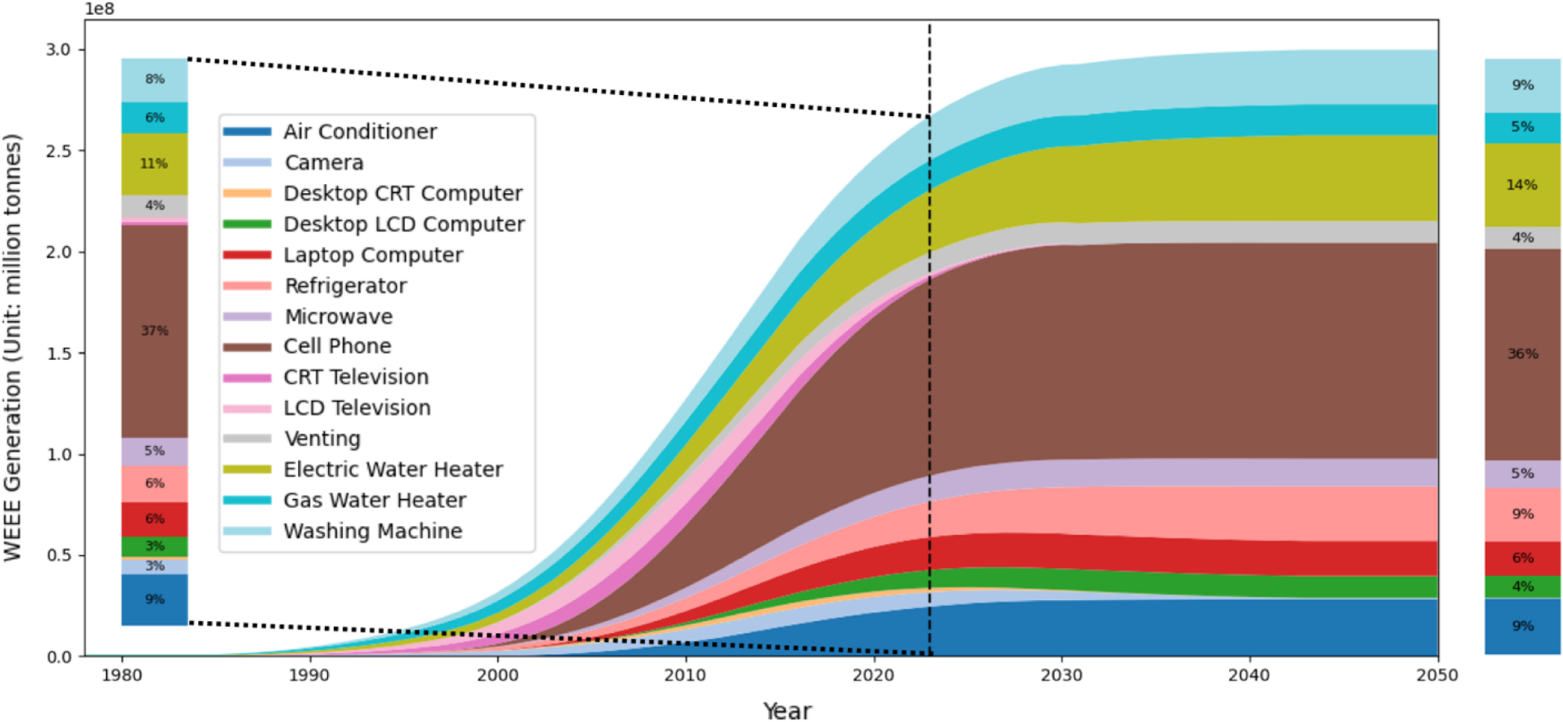
Historical (1978–2023) and projected (2024–2050) national household WEEE generation in China by product type. The stacked area plot shows annual WEEE generation (million units) for the 14 appliance types, with the dashed vertical line indicating the transition from historical reconstruction to projection. The left inset magnifies the historical period, and the side bars display the composition shares of each product type in total WEEE generation in 1990 and 2050, respectively.

**Figure 5:**
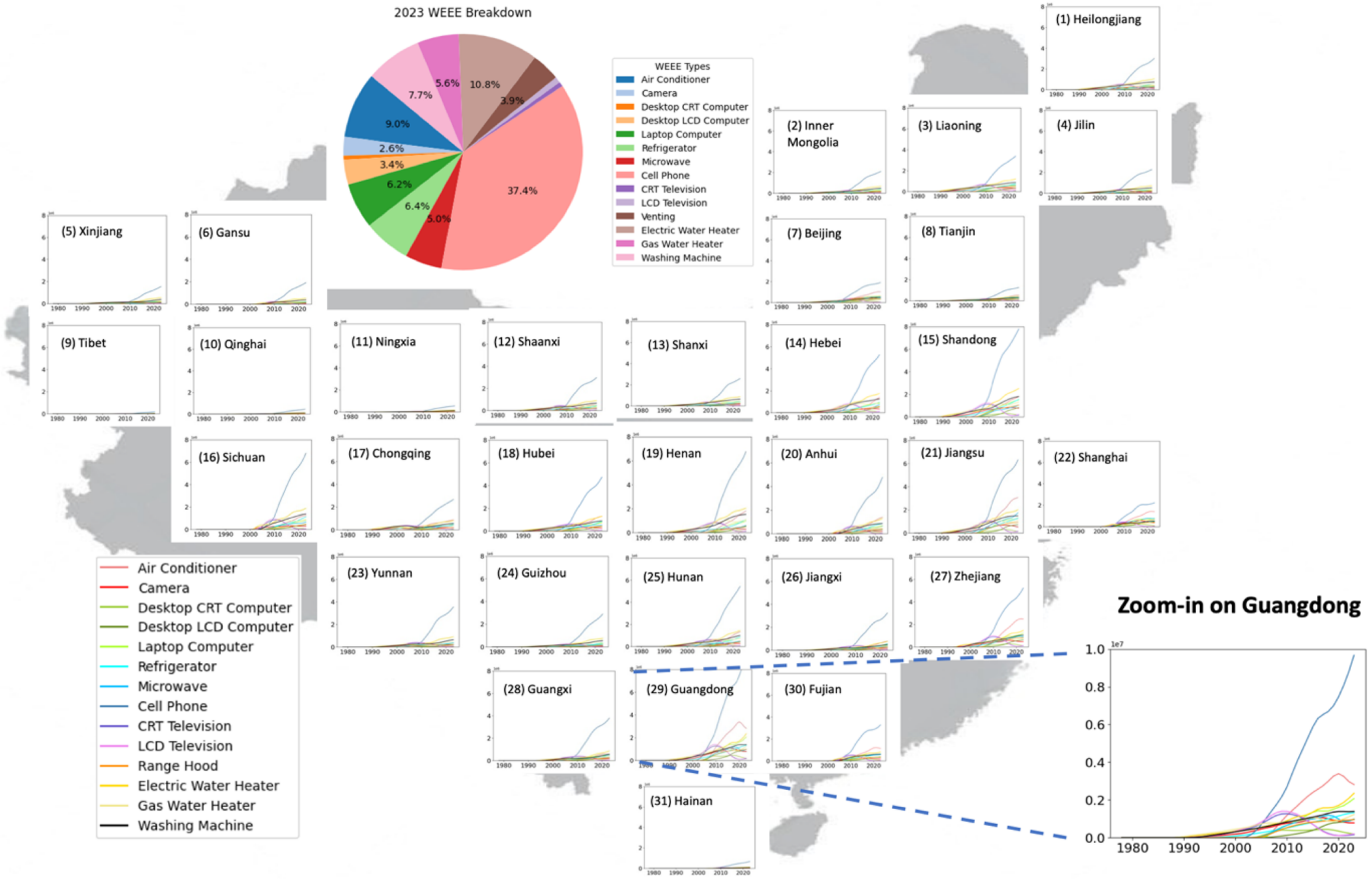
Provincial-level WEEE generation (in units) from 1978 to 2023.

Nationally, household WEEE generation remained relatively low before the early 1990s and then increased rapidly with the expansion of appliance ownership. Total WEEE generation exceeded 100 million units in 2008 and has grown steadily since then. By 2023, China generated more than 400 million units of household WEEE, including over 26 million air conditioners, about 30 million computers, around 19 million refrigerators, more than 4 million televisions, and over 22 million washing machines. These magnitudes and trends are in line with previous national assessments of household WEEE in China, although our estimates cover a broader set of product types and a longer time horizon.^62^

The composition of WEEE by product type has also evolved over time. In the late 1990s and early 2000s, televisions, refrigerators, and washing machines dominated household WEEE generation, reflecting earlier phases of diffusion for large appliances. With the rapid uptake of cell phones and personal computers since the 2000s, information and communication devices have become increasingly important contributors. By 2023, cell phones constitute the largest single source of WEEE in terms of unit counts, owing to their near-saturated penetration and short replacement cycles. When cell phones are excluded, total WEEE generation first surpasses 100 million units in 2011 and rises to around 180 million units by 2023, driven mainly by the maturation and replacement of large appliances and the continued diffusion of computers and other small electronics.

Pronounced regional heterogeneity is observed in the spatial distribution of WEEE generation (Figure 5). Eastern and southeastern coastal provinces such as Guangdong, Jiangsu, Zhejiang, and Shandong have been persistent hotspots of household WEEE generation since the 1990s, reflecting early and widespread adoption of appliances as well as higher incomes and faster replacement. Several rapidly urbanizing central provinces (e.g., Hubei, Hunan, Anhui) show a delayed but steep increase in WEEE generation starting in the 2000s, corresponding to a catch-up phase in both ownership and replacement. Western provinces generally exhibit lower levels of WEEE generation, but their output has accelerated in the past decade as appliance penetration expands and first-generation devices approach the end of life. These differentiated provincial trajectories imply that peaks in WEEE generation will occur at different times across regions and that high-volume WEEE flows are already concentrated, and will likely remain so in the near term in coastal and southeastern China. This spatial heterogeneity foreshadows potential mismatches between where WEEE is generated and where formal recycling capacity is currently located.

To explore the long-term evolution of household WEEE, Figure 4 also presents the projected evolution of total national household WEEE generation by product type up to 2050. The projections indicate that total WEEE generation in China will continue to increase over the next two to three decades, although the growth rate gradually slow as appliance ownership approaches saturation. National WEEE generation is expected to rise most rapidly up to around the 2030s and then level off toward midcentury, reflecting a transition from a diffusion-driven phase to one dominated by replacement of existing stocks. At the same time, the composition of WEEE continues to shift. Cell phones remain the largest single contributor throughout the projection period, accounting for roughly one-third to two-fifths of total units by 2050. Large household appliances such as air conditioners and washing machines, together with computers, make up most of the remaining volume, while legacy technologies such as CRT televisions disappear almost entirely from the waste stream, and LCD televisions gradually lose relative importance.

In addition to the aggregate national projections, we further examine the product-specific generation trajectories for all 14 appliance types (Figure S3). In particular, the timing of peak WEEE generation differs markedly across product types: legacy technologies such as CRT televisions have already passed their peak and decline rapidly toward negligible levels before 2035, whereas LCD televisions and several computer types are projected to peak later, around the 2030s, and large appliances such as refrigerators and washing machines show more gradual approaches to saturation without a pronounced peak. The steepness of the trajectories also varies, with mobile phones and other information and communication technologies exhibiting relatively concentrated retirement waves, while large appliances display more spread-out retirement profiles. Overall, the projections suggest that, in the absence of major changes in product lifetimes or consumption patterns, China’s household WEEE flows will remain substantial and increasingly concentrated in a limited number of high-volume categories. Meanwhile, differences in peak timing are critical for anticipating when specific product types will exert the greatest pressure on collection and recycling systems, with significant implications for how these systems should be designed and scaled in the future.

### 3.3 AM Reserve Estimation

To quantify the resource recovery potential of household WEEE, we further estimate the theoretical AM reserves embedded in household WEEEs. Figure 6 provides an overview of the annual total AM reserves from 1978 to 2023. Unsurprisingly, base metals and associated nonmetallic materials account for by far the largest share of these reserves, with steel, plastics, and copper dominating in mass terms. By 2023, steel and plastics each exceed 2 million tonnes and 1 million tonnes, respectively, while copper reserves reach about 228 thousand tonnes, equivalent to roughly 9.4% of China’s recycled copper volume in that year (Figure 6a).^63^ For economically valuable metals, lead (Pb) is the most abundant, followed by tin (Sn) and zinc (Zn) (Figure 6b). By 2023, the cumulative Pb reserves in household WEEE amount to several tens of thousands of tonnes, whereas Sn and Zn each reach the order of a few thousand tonnes. Precious and critical metals including cobalt (Co), gold (Au), silver (Ag), indium (In), palladium (Pd), and ruthenium (Ru) are present in much smaller tonnages, typically from a few hundred kilograms to a few tens of tonnes, but with very high unit values (Figure 6c). Among these, cobalt forms by far the largest stock in mass terms, exceeding 1,000 tonnes by 2023. Meanwhile, gold reserves in household WEEE exceed 21 tonnes (about 5.6% of China’s annual primary gold production^64^), and palladium reserves are around 7 tonnes (approximately 50% of the country’s primary Pd output^65^), underscoring the strategic importance of WEEE as a secondary source of both bulk and high-value metals.

**Figure 6:**
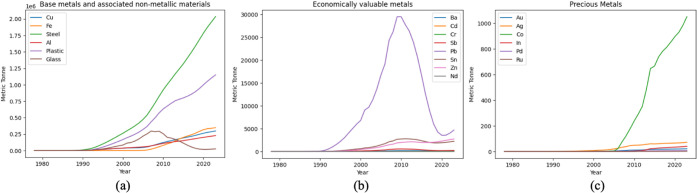
Annual AM reserves embedded in household WEEE in China, 1978–2023: (a) base metals and associated nonmetallic materials; (b) economically valuable metals; (c) precious and critical metals (tonnes).

The buildup of steel, plastics, and a large share of copper in the AM reserves is primarily associated with the rapid expansion and subsequent retirement of large household appliances such as refrigerators, washing machines, air conditioners, and water heaters, whose WEEE generation trajectories show sustained growth from the 1990s onward (Figure 4 and Figure S4). In contrast, the sharp increase in copper and several precious/critical metals (e.g., Co, Au, Pd, In) since the 2000s also reflects the proliferation and relatively short lifetimes of information and communication technologies (Figure S4), most notably cell phones and laptop computers, whose retirement waves overlap with those of large appliances. Pb- and glass-bearing WEEE display a different pattern: both reserves rise from about 1990 to 2010 and then decline sharply thereafter, reflecting the phase-out of CRT televisions, in which leaded glass is a major component of the display unit.

Figure 7 further illustrates the spatial distribution and evolution of AM reserves for selected key metals, i.e., steel, Al, Cu, Pb, Au, and Co in 2013, 2018, and 2023. Across all six metals, a pronounced and persistent east–west gradient is evident. Metal-rich WEEE is concentrated along the Bohai Rim (Beijing-Tianjin-Hebei, Liaoning), Inner Mongolia, and the Yangtze River Delta (Jiangsu-Shanghai-Zhejiang), while most western and southwestern provinces (e.g., Gansu, Qinghai, Xinjiang, Tibet, Yunnan) remain at low levels throughout the period. Within this overall pattern, Au- and Co-bearing WEEEs are the most tightly clustered, with reserves peaking in a small group of provinces (Inner Mongolia, Jilin, Hebei, Henan); Steel, Al, and Cu have a slightly broader but still clearly coastal-biased footprint, indicating that structural and casing materials are more evenly distributed than precious metals. Temporally, from 2013 to 2023, the eastern high-reserve corridor for most metals deepens and gradually extends inland, especially for steel, Al, Cu, and Co. In contrast, the intensity of Pb reserves declines over time in many of the provinces that were initially high, particularly in traditional CRT-TV regions in the east and northeast, leading to a weakening and fragmentation of the original Pb hotspot pattern by 2023. Consequently, regionally differentiated collection and recycling strategies, such as prioritizing eastern and emerging inland hotspots, are likely to be more effective than uniform, nationwide approaches.

**Figure 7:**
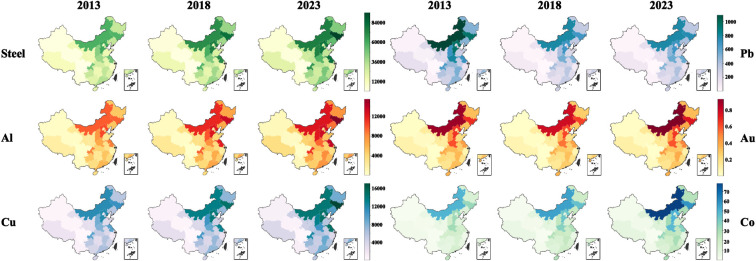
Provincial metal content in household WEEE in China for selected years (2013, 2018, 2023).

### 3.4 Recycling Gap Estimation

Building on the spatiotemporal patterns of WEEE generation and AM reserves described above, we next examine how much of these flows are actually captured and how this varies across product categories and provinces. Due to the lack of WEEE type-resolved information for illegally imported WEEE, this paper focuses exclusively on domestic household-generated WEEE and its treatment by certified recycling enterprises. Figure 8 presents the cumulative WEEE that is recycled versus unrecycled for each category between 2013 and 2023 of the 109 licensed dismantling enterprises for five regulated categories, i.e., air conditioners, computers, refrigerators, washing machines, and televisions.

**Figure 8:**
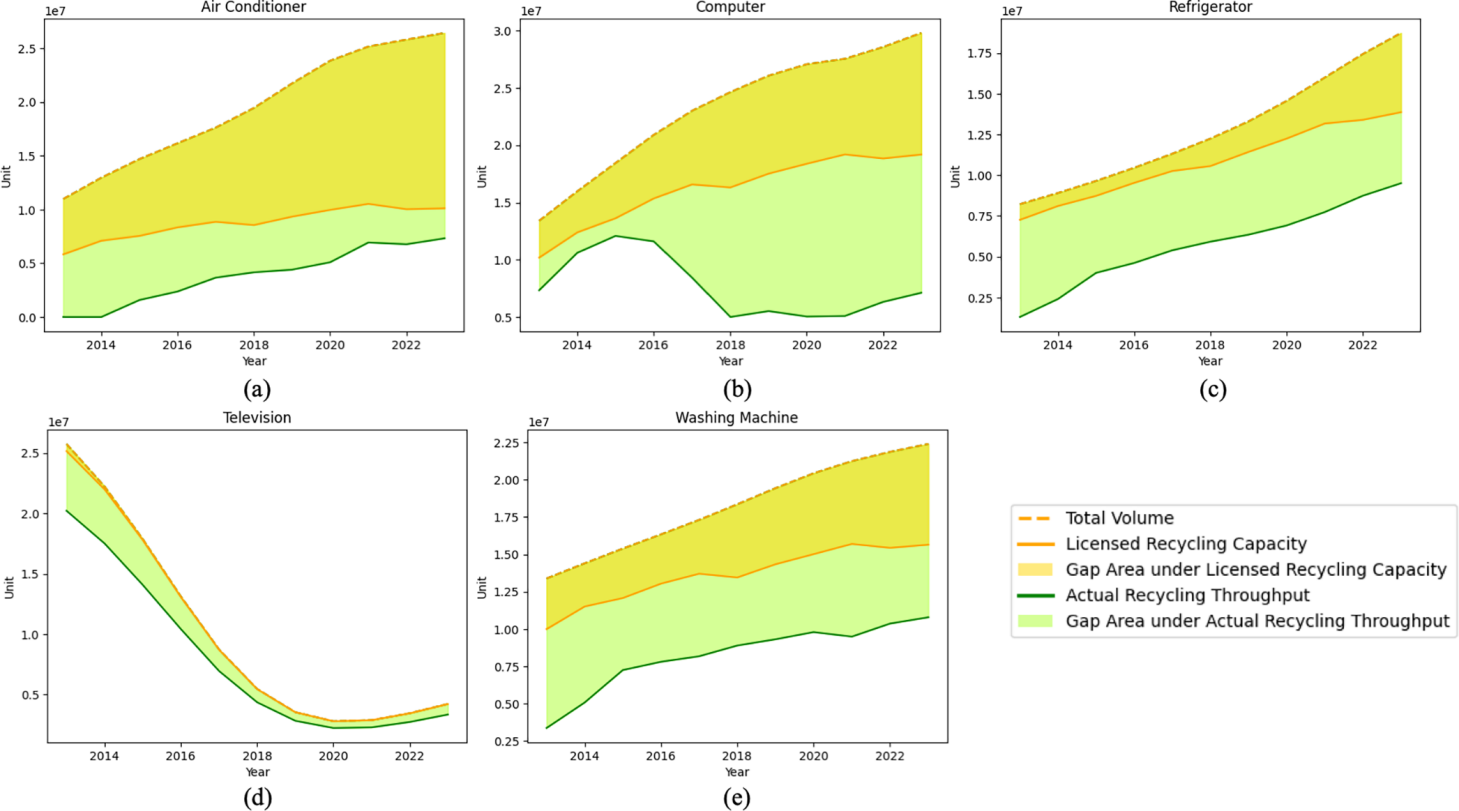
Recycled vs unrecycled WEEE from 2013 to 2023. The dashed line denotes total WEEE generation, while the green and yellow solid lines show the recycled volumes under the licensed-capacity and actual-operation scenarios. The shaded areas between the generation curve and the recycling curves represent WEEE that is not treated by certified facilities.

As indicated by Figure 8, both licensed capacities and actual recycling volumes have increased over time, reflecting expanded efforts for formal WEEE recycling. However, clear discrepancies and systemic mismatches emerge across product categories. For computers, refrigerators, and washing machines, persistent shortfalls are observed, with actual recycling volumes remaining well below licensed capacities throughout the period, and the gap widening markedly for computers after 2018 as certified plants are increasingly under-utilized. Air conditioners and televisions occupy an intermediate position, where licensed capacities are generally closer to realized operation levels, and the unrecycled volumes implied by the difference between the two curves are relatively smaller and more stable over time.

When we take a further look at the total WEEE generation, under the licensed capacity, less than 10% of end-of-life televisions remained unrecycled, whereas under actual operation, 25–35% of televisions are not recovered by certified facilities. This gap indicates that, for televisions, the existing network of licensed enterprises is in principle large enough to handle most of the flow, but collection performance and facility utilization are insufficient to achieve that potential. Similarly, for refrigerators, the licensed capacity is already close to what would be needed to substantially reduce unrecycled volumes, yet realized recycling still leaves a noticeably larger share unrecovered, again pointing to operational rather than structural constraints. In contrast, for air conditioners, computers, and washing machines, even the licensed capacities are clearly inadequate. Throughout the period, ∼55% of generated WEEEs in these categories remain unrecycled under the licensed-capacity scenario, and this share increases to roughly 70% under actual operation in 2023. The gap widens toward the end of the time series as WEEE generation from these appliances accelerates, suggesting that capacity expansion has not kept pace with the growth of end-of-life flows or systematic weaknesses in collection networks for these product types. For computers and televisions in particular, which contain relatively high concentrations of precious and critical metals, the persistent under-recycling implies that a large fraction of high-value anthropogenic minerals is currently lost to informal treatment or disposal.

For each metal category, as WEEE generation increases over time, the theoretical recoverable amounts of almost all metals grow steadily (Figure 9). Similarly, there is a persistent and often large gap between actual recycling volumes and both the theoretical content and the licensed-capacity scenario. Under actual operation, more than 70% of the most valuable precious metals (Au, Ag, In, Pd, Ru, Nd) embedded in household WEEE are not recovered in any given year. Even under full utilization of licensed capacity, capture rates for these precious metals remain far from complete, indicating that the current formal system is still unable to fully exploit their high economic potential. For economically valuable metals such as Pb, Sn, and Zn, realized recovery typically hovers around 40–50% of the theoretical content, with some elements such as Ba and Cr exhibiting particularly low capture rates of only about 20%.

**Figure 9:**
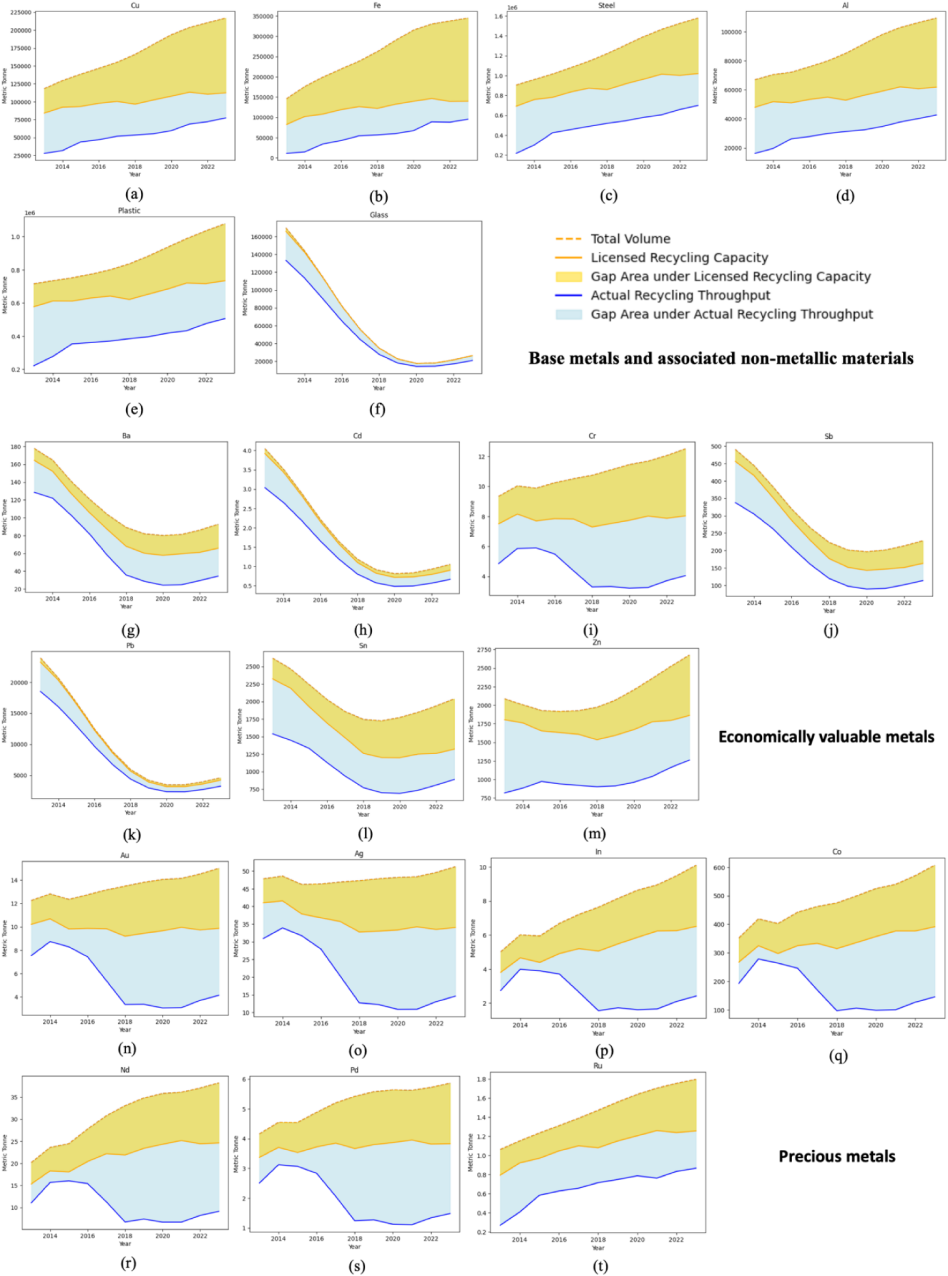
Recycled volumes of metals from household WEEE in China under actual-operation and licensed capacity, 2013–2023 (tonnes). Shaded areas denote unrecovered metal relative to total content in WEEE.

Base metals are recovered in larger absolute quantities but still with substantial losses. Under actual operation, only about 30–50% of Cu, Fe, and steel contained in household WEEE is captured; even if all certified facilities operated at their licensed capacities, the recovery rates of Cu and Fe would rise only to around 50% and 60%, respectively, leaving a significant share of these bulk metals unrecovered. Overall, improving operational performance to approach licensed capacity would yield considerable gains for most metals, but for several high-value and base metals, additional capacity and more effective collection are required to close the gap between theoretical AM reserves and realized recovery.

Beyond category-specific gaps, pronounced spatial mismatches also emerge when comparing where AM reserves are located with where licensed capacity and actual recycling are concentrated (Figure S5). The 2023 maps for steel, Al and Cu show that metal-rich WEEE is clustered along the Bohai Rim and the Yangtze River Delta, yet licensed capacity and realized recycling are even more tightly concentrated in a subset of these provinces, leaving several inland provinces with growing AM reserves but limited formal treatment. For Au and Co, the contrast is sharper. Reserves are high in a small group of northern and central provinces, whereas much of the licensed capacity and actual throughput are located in coastal hubs, indicating that high-value flows either travel long distances or are partly captured by informal channels. For Pb, associated mainly with CRT-related WEEE, both reserves and recycling shift over time, with some traditional hotspots showing declining reserves but still hosting a disproportionate share of treatment capacity. Taken together, these spatial patterns confirm that current infrastructure does not systematically align with the provincial distribution of AM reserves, reinforcing the need for region-specific capacity planning and interprovincial logistics arrangements.

Using the average of the 5-year maximum and minimum nominal market prices for tradable metals (Table S3), we estimate the theoretical gross value (without considering collection, processing, and compliance costs) of metals recovered from household WEEE under the actual-operation and licensed-capacity scenarios. In 2023, metals recovered under actual operation correspond to a theoretical gross value of roughly 2 billion USD, whereas full utilization of the existing licensed capacity could increase this value to about 5 billion USD (Figure S6). The gap between the licensed-capacity value and the theoretical maximum value embedded in WEEE is even larger, underscoring the magnitude of unrealized economic potential. More details for the composition of recycling value for each metal can be found in Figure S7.

### 3.5 Sensitivity Analysis

Figure 10 summarizes the sensitivity of our WEEE estimates to both product-level Weibull parameters and regional lifetime heterogeneity. We first examine the relative influence of the scale (*μ*) and shape (σ) parameters using CRT televisions as an example (Figure 10a,b). Under ± 10% perturbations, deviations from the baseline are negligible before the early 1990s, when stocks and retirements are still small. From roughly 1990 to 2015, when CRT adoption and retirement accelerate, changes in *μ* and σ begin to matter. Perturbing *μ* mainly shifts the timing and height of the retirement wave: a lower *μ* leads to an earlier and higher peak in WEEE generation, whereas a higher *μ* delays and slightly reduces the peak. Perturbing σ, by contrast, has only a limited effect on the peak year and primarily changes the spread of retirements around the peak, with noticeable but short-lived deviations concentrated around 2008–2012. After 2015, all trajectories reconverge as CRT stocks are exhausted. These results confirm that *μ* effectively controls the average service life and exerts a stronger influence on WEEE generation than σ, which mainly governs the dispersion of failures.

**Figure 10:**
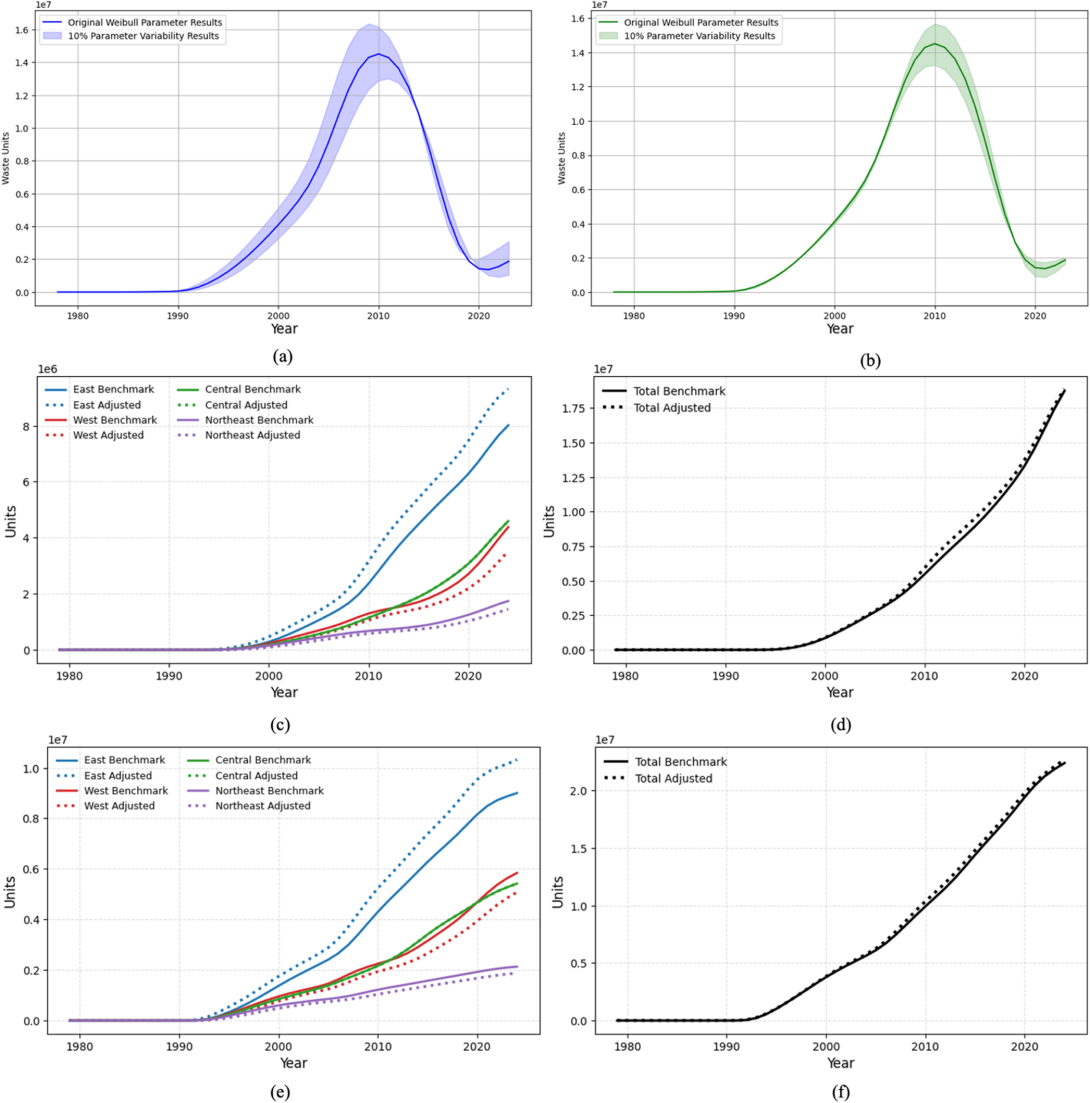
Sensitivity analysis of household WEEE generation to Weibull parameters and regional lifetimes. (a, b) Product-level sensitivity for CRT TVs under ±10% changes in the Weibull scale *μ* (a) and shape σ (b). (c–f) Regional sensitivity for refrigerators (c, d) and washing machines (e-f), comparing baseline and regional-lifetime scenarios (*μ*–10% in eastern, 0% in central, +10% in western and northeastern provinces; σ fixed) at regional (c, e) and national (d, f) levels.

Given the dominant role of *μ* in shaping WEEE trajectories, we then use *μ* as the lever to explore the effects of regional lifetime heterogeneity. Panels 10­(c–f) present the regional sensitivity analysis for washing machines and refrigerators. Following the National Bureau of Statistics classification, we group provinces into eastern, central, western and northeastern regions and construct a regional-lifetime scenario in which *μ* is decreased by 10% in eastern provinces, kept unchanged in central provinces and increased by 10% in western and northeastern provinces, while σ is held constant. For washing machines (Figure 10c), this adjustment yields slightly higher and earlier WEEE generation in the east and correspondingly lower and later flows in the west and northeast. Refrigerators show a similar pattern (Figure 10e). In both cases, however, the rank order of regional contributions does not change: the east remains the largest source of WEEE, followed by the central region, with the west and northeast contributing smaller shares in both scenarios. At the national level (Figure 10d,f), the baseline and adjusted trajectories are nearly indistinguishable, indicating that plausible regional variations in *μ* redistribute WEEE flows across regions but have only modest effects on national totals.

## 4 Discussion

This study provides a comprehensive analysis of WEEE generation, recycling, and the potential for AM recovery in China from 1978 to 2023, with a focus on household appliances. Leveraging provincial-level data and the SMFA method, our research reveals several critical findings: (1) both in-use EEEs and WEEE generation have shown steady growth over the past decades; (2) the current recycling capacity is insufficient for effective metal resource recovery, with particularly imbalanced allocation across WEEE types and geographical locations; (3) the recycling rates for economically valuable and precious metals are still low. This study provides the longest temporal and most granular spatial analysis of household WEEE in China to date, and explicitly quantifies province-level mismatches between WEEE/AM generation and licensed as well as realized recycling volumes. These features allow us not only to characterize where and when household WEEE and AM reserves accumulate, but also to identify where the current system fails to transform these secondary resources into formal recovery.

### 4.1 Limitations and Future Research

Several limitations should be acknowledged when interpreting our results. First, the SMFA framework and Weibull lifetime modeling employed here are based on widely used and empirically validated approaches. We deliberately chose these methods to ensure transparency, comparability with earlier studies and robustness over time, and place the main innovation of this work in the way these established tools are combined with long time series, province-resolved data, detailed product categorization, and firm-level recycling information, rather than in proposing a new functional form for product lifetimes. At the same time, this choice implies that uncertainties in lifetime parameters and future saturation levels remain an important limitation. Although our sensitivity analysis suggests that moderate variations in lifetime parameters do not overturn the main patterns, changes in product lifetimes, market saturation, and technology shifts (for example, new device types or design changes) may affect the projected WEEE and AM trajectories beyond 2030. Future work could explore alternative lifetime formulations, probabilistic calibration, or data-driven approaches to further test and refine these projections.

Second, our recycling gap analysis is restricted to household WEEE treated by certified facilities and to the five regulated categories for which consistent capacity and operation data are available. Flows handled entirely in informal channels, as well as WEEE from commercial and industrial users, are not explicitly modeled and may be substantial in some provinces. As a result, the “unrecycled” WEEE quantified in this study should be interpreted as material that is not processed by certified dismantling enterprises, rather than as flows for which no recovery occurs at all. In practice, a non-negligible share of these “unrecycled” units, particularly large household appliances and information and communication devices, may be collected and dismantled by informal actors, which would reduce the true gap between theoretical AM reserves and total metals actually recovered. However, existing studies also indicate that informal recycling often operates with lower material recovery efficiencies and weak environmental and occupational safeguards, so that metals are more likely to be lost or dispersed and hazardous substances are more likely to be released. In this sense, the recycling gaps reported here can be regarded as conservative upper bounds on the shortfall between theoretical AM availability and formal recovery under environmentally regulated conditions. As more than 60% of the WEEEs are processed through informal channels, extending the integrated stock-flow-capacity framework developed in this paper to include additional streams, and to better quantify the scale and performance of informal recycling, would provide a more complete picture of AM recovery and help to clarify where integrating or transforming informal activities could yield the greatest environmental and resource benefits.

Third, our SMFA relies on historical statistics for appliance ownership, average weights, and material compositions, which are partly incomplete and had to be interpolated or approximated from a limited set of benchmark years. While we have used the best available sources and cross-checked ranges with previous studies, these data gaps introduce additional uncertainty into the historical reconstructions and long-term projections. Beyond data limitations, the projections themselves depend on structural assumptions about how appliance markets, technologies, and policies will evolve. The logistic and Hubbert-type functions used to extrapolate WEEE generation implicitly assume that past diffusion and phase-out patterns continue under broadly similar socio-economic conditions. In reality, changes in ownership saturation levels, shifts in consumer behavior (for example, increased repair, reuse or “right-to-repair” uptake), new device types and design concepts, and stronger policy interventions such as extended producer responsibility or eco-design regulations could either accelerate or delay replacement cycles and alter both the magnitude and composition of future WEEE and embedded metals. Our lifetime sensitivity analysis indicates that moderate parameter variations do not overturn the main spatial patterns identified in this study, but fully capturing such structural uncertainties will require explicit scenario analysis that couples alternative policy and technology pathways with the SMFA framework. Future work could therefore build on the present model by combining improved, more frequent reporting of product characteristics and composition with scenario-based explorations of “business-as-usual”, “lifetime extension” and “high-circularity” pathways, in order to better bound the range of plausible WEEE and AM trajectories toward midcentury.

Fourth, a further limitation is that the Weibull lifetime parameters used in this study are derived from national-level literature and, in the baseline setting, are applied uniformly across all provinces and between urban and rural households. Empirical analyses based on recycling-plant surveys^60^ show that actual service lifetimes can differ systematically across regions, with shorter lifetimes in some eastern and central areas and longer lifetimes in northern and northeastern China. Our macro-regional sensitivity analysis, in which eastern lifetimes are shortened and western lifetimes extended, suggests that such heterogeneity changes the magnitude of provincial WEEE and AM estimates but does not alter the main spatial patterns of WEEE and AM accumulation or the associated policy implications. Nonetheless, as more detailed, province-specific lifetime data become available for a broader range of product types, the SMFA framework developed here can be further calibrated to capture regional differences in usage, repair, and replacement behavior more precisely.

Finally, our study focuses on the quantity and economic value of recoverable materials, but does not quantify environmental impacts or cobenefits, such as avoided primary mining, energy use, or emissions. Future research could link the present provincial-level WEEE and AM estimates with life-cycle assessment and scenario analysis, and incorporate behavioral drivers of collection and disposal, to evaluate how alternative policy and technology options would influence both resource efficiency and environmental outcomes.

### 4.2 Implications for the Design of WEEE Recycling Systems

Despite these limitations, the results point to several priorities for improving China’s WEEE recycling system. The large and persistent gap between theoretical AM reserves and the amounts recovered by certified enterprises indicates that system performance is constrained less by a lack of facilities than by misalignment between where WEEE arises, where capacity is located, and how effectively flows are collected and channeled. Addressing this mismatch requires both a more balanced spatial layout of infrastructure and more effective use of existing plants. In coastal and southeastern provinces, where household WEEE and AM reserves are already concentrated and licensed capacities are relatively high but often under-utilized, particularly for televisions and computers, strengthening collection networks, retailer take-back schemes, and integration with municipal waste services is likely to yield larger benefits than further capacity expansion. In contrast, several rapidly urbanizing inland provinces show steeply rising WEEE flows but comparatively limited licensed capacity and weaker collection infrastructures. For these emerging hotspots, forward-looking infrastructure planning and the development of regional treatment hubs could prevent a growing share of WEEE from being diverted into informal treatment. High-loss categories such as air conditioners and washing machines, for which even licensed capacities are clearly insufficient, merit dedicated collection and recycling programs that explicitly target their large and growing end-of-life streams.

From a national perspective, a hub-and-spoke configuration could help formalize the existing spatial concentration of WEEE flows. The Bohai Rim (Beijing-Tianjin-Hebei-Shandong) and the Yangtze River Delta (Jiangsu-Shanghai-Zhejiang) could serve as core processing hubs, with surrounding provinces such as Henan, Anhui, Hubei, and Guangdong attached as catchment zones through subsidized reverse-logistics and streamlined cross-provincial transport permits. Within such a structure, high-value recycling lines for Au-, Pd-, Co-, and Cu-rich streams from information and communication equipment could be colocated with metal refining capacity in the hubs, while bulk lines for Al and steel are integrated with smelters in industrial provinces such as Hebei, Shandong, Jiangsu, and Zhejiang. System performance could be further improved by moving from static, capacity-based licensing to performance-oriented permitting that rewards high utilization, metal-specific recovery yields and strict compliance, especially for hazardous residues such as Pb-containing fractions. A national electronic manifest system that tracks WEEE units from collection to final metal outputs and discloses key performance indicators at the provincial level would increase transparency and allow targeted incentives, for example, throughput bonuses where capacity is under-used, logistics credits for moving material from inland and western provinces, and EPR-funded floor contracts for recovered metals to reduce revenue volatility.

### 4.3 Region-Specific Recommendations for Policymakers, Industry, and Other Stakeholders

The province-level WEEE and AM maps generated in this study also provide a basis for more differentiated interventions by policymakers, industry, and other actors. At the national and provincial levels, collection and recycling targets could be adjusted to reflect local WEEE and AM profiles, with higher formal collection and recovery goals for provinces along the Bohai Rim and Yangtze River Delta where high-value metals such as copper, cobalt, and precious metals are particularly concentrated. Financial support schemes and extended producer responsibility arrangements can be calibrated accordingly, for example by directing stronger incentives toward provinces where projected WEEE peaks are imminent but formal capacity is still insufficient, and by linking subsidies more closely to metal-specific recovery rather than simple throughput. Beyond infrastructure and incentives, strengthening EPR obligations for producers and improving standardized data reporting by recycling companies would enhance accountability and facilitate medium- to long-term planning.

For industry and technology stakeholders, integrating informal collectors into formal channels is essential to improve collection efficiency and expand coverage, especially in rural and peri-urban areas. Partnerships between certified dismantling enterprises and informal networks, supported by simplified licensing and standardized buy-back prices, can help capture more appliances that currently bypass formal systems. At the same time, consumer incentive programs, such as trade-in schemes, deposit-refund systems or small rebates for returning specific product types, can encourage households to release dormant devices and return end-of-life products to regulated channels. On the technological side, investment should focus on upgrading recycling processes to raise recovery rates for both bulk metals like copper and steel and precious metals such as gold and palladium, many of which remain largely unrecovered despite their high economic and environmental value. Region-specific specialization is also important: advanced facilities in coastal hubs can concentrate on high-value consumer electronics such as mobile phones and computers, whereas inland plants may emphasize large household appliances, including air conditioners and washing machines, that dominate local WEEE streams. In parallel, manufacturers can support system performance through design-for-disassembly and modular product design that facilitate repairs, upgrades and high-quality material recovery, thereby reducing premature disposal and enhancing the overall circularity of household appliances.

## Supplementary Material



## Data Availability

The data that support the findings of this study are available from the corresponding author upon reasonable request.

## Notes

Household appliance subtype market shares (Table S1); Weibull lifetime parameters for 14 appliance types (Table S2); metal market prices and currency/unit conversions (Table S3); WEEE unit weights by product type (Table S4); time-varying WEEE unit weights (Figure S1); type and provincial breakdown of recycled WEEE and licensed/actual capacities (Figure S2); product-specific WEEE generation trajectories for 14 appliance types, 1978–2050 (Figure S3); metal contents in different WEEE types and circuit boards (Figure S4); provincial metal-specific recycling gaps (Figure S5); economic value of recovered metals under actual-operation and licensed-capacity scenarios (Figure S6); temporal composition of recycling value by metal (Figure S7); parameter and regional sensitivity analyses (Figure S8); historical WEEE imports (Figure S9); population and appliance ownership data processing (Text S1); reconstruction of WEEE imports (Text S2); and temporal evolution and metal composition of recycling value (Text S3) (PDF)
