# Supplementary material for: Spatiotemporal Distribution of Household WEEE and Anthropogenic Mineral Reserves in China from 1978 to 2050
Source: Environ Sci Technol. 2026 Mar 25;60(15):11446–62. doi: 10.1021/acs.est.6c02246 (PMC13104169; doi:10.1021/acs.est.6c02246)
Supplement: Supplementary file 1 [file es6c02246_si_001.pdf]

Supplemental Information for

Spatiotemporal distribution of household WEEE and anthropogenic mineral reserves in China from 1978 to 2050

Zongqi Yu[1], Yifan Gu[2], Chenyang Shuai[3], Xi Chen[4], Ming Xu[5], Bu Zhao[6,*]

[1] Tepper School of Business, Carnegie Mellon University, Pittsburgh, Pennsylvania 15213, United States

[2] Institute of Circular Economy, College of Materials Science and Engineering, Beijing University of Technology, Beijing 100124, P.R. China

[3] School of Management Science and Real Estate, Chongqing University, Chongqing 400044, P.R. China

[4] College of Economics and Management, Southwest University, Chongqing 400715, P.R. China

[5] School of Environment, Tsinghua University, Beijing 100084, P.R. China

[6] Department of Environmental and Sustainable Engineering, University at Albany, State University of New York, Albany, New York 12222, United States

***Corresponding Author:** Bu Zhao, Department of Environmental and Sustainable Engineering, University at Albany, State University of New York, Albany, New York 12222, United States

Email: bzhao@albany.edu

This PDF file includes:

Table S1-4
Figure S1-4
Text S1-2

Table S1: In-Use Home Appliance Sub-Type Market Share

Television (CRT vs. LCD)

	1999	2000	2001	2002	2003	2004	2005	2006	2007	2008	2009	2010
LCD	0	0.05	0.1	0.2	0.3	0.4	0.5	0.6	0.7	0.8	0.9	1
CRT	1	0.95	0.9	0.8	0.7	0.6	0.5	0.4	0.3	0.2	0.1	0

Computer (Desktop vs. Laptop)

	2000	2001	2002	2003	2004	2005	2006	2007	2008	2009
Desktop	1	1	1	1	0.649	0.639	0.6	0.582	0.593	0.548
Laptop	0	0	0	0	0.351	0.361	0.4	0.418	0.407	0.452

2010	2011	2012	2013
0.569	0.573	0.583	0.552
0.431	0.427	0.417	0.448

Desktop Computer (CRT vs. LCD)

	1999	2000	2001	2002	2003	2004	2005	2006	2007	2008	2009
LCD	0	0.05	0.1	0.15	0.2	0.25	0.3	0.35	0.4	0.45	0.5
CRT	1	0.95	0.9	0.85	0.8	0.75	0.7	0.65	0.6	0.55	0.5

2010	2011	2012	2013	2014	2015	2016	2017	2018	2019
0.55	0.6	0.65	0.7	0.75	0.8	0.85	0.9	0.95	1
0.45	0.4	0.35	0.3	0.25	0.2	0.15	0.1	0.05	0

Water Heater (Electrical vs. Gas)

	2001	2002	2003	2004	2005	2006	2007	2008	2009	2010	2011
Electrical	0.43	0.45	0.55	0.5	0.49	0.57	0.61	0.61	0.51	0.58	0.61
Gas	0.57	0.55	0.45	0.5	0.51	0.43	0.39	0.39	0.49	0.42	0.39

2012	2013	2014	2015	2016	2017	2018	2019	2020	2021	2022	2023
0.69	0.74	0.7	0.71	0.7	0.71	0.7	0.71	0.7	0.71	0.71	0.7
0.31	0.26	0.3	0.29	0.3	0.29	0.3	0.29	0.3	0.29	0.29	0.3

Table S2: Weibull Parameters

	Scale	Shape
AC	12.3	2.8
CAM	8.2	1.4

FR	16.5	2.6
MW	10.126	2.655
PHONE	7.6	1.7
TV-CRT	12.6	2
TV-LCD	12	2.1
VT	13.5	2
WH-EWH	7.9	1.8
WH-GWH	7.9	1.8
WM	13.9	2.2
CP-Desk (CRT, LCD)	9.6	2.1
CP-Laptop	5.2	1.5

Table S3: Metal Market Prices

In the following tables, we used the following values for converting the units:

1. CNY to USD has 5-year high of 7.365 to 1, and 5-year low of 6.3221 to 1. Here, we use the average value between 5-year high and low, being 6.844 to 1.
2. LB to Tonne: 2204.62 to 1
3. T.oz to Tonne: 32150.7466

**Basic Material**	5-year High	5-year Low	Average	Price at USD/Tonne
Cu	5.05	2.16	3.605 USD/LB	7947.66
Fe	90.79	219.77	155.28 USD/Ton	155.3
Steel	5925	2880	4402.5 CNY/Ton	643.31
Al	3849	1453	2651 USD/Ton	2651
Plastic	/	/	/	/
Glass	/	/	/	/

**Metal and Rare Earth**	5-year High	5-year Low	Average	Price at USD/Tonne
Ba	/	/	/	/
Cd	/	/	/	/
Cr	/	/	/	/
Sb	/	/	/	/
Pb	2461.8	1579.8	2020.8 USD/T	2020.8
Sn	47540	13633	30586.5 USD/T	30586.5
Zn	4434.5	1846.5	3140.5 USD/T	3140.5
Nd	/	/	/	/

**Precious Metal**	5-year High	5-year Low	Average	Price at USD/Tonne
Au	2747.6	1497.3	2122.45 USD/t.oz	68238352.1
Ag	33.7	12.609	23.1545 USD/t.oz	744434.5
Co	82000	24300	53150 USD/T	53150
In	3075	925	2000 CNY/kg	292246.0
Pd	3002.4	859.15	1930.775 USD/t.oz	62075857.8
Ru	/	/	/	/

Figure S1: WEEE Unit Weight

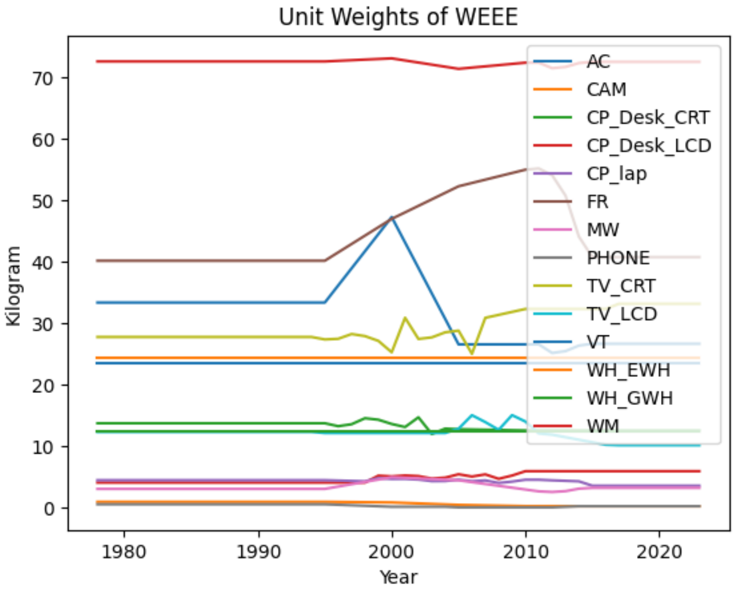

Figure S2: WEEE Type and Provincial Breakdown in Recycling

In the analysis, both the actual capacity and the designed capacity are disclosed by from the white papers disclosed by Extended Producer Responsibility Technology and Innovation Alliance. What is worth noting here, is that the government regulated recycling covers only AC, CP, FR, TV, and WM.

In the analysis using actual recycling capacity, the actual recycling level is obtained from the white papers disclosed by (Extended Producer Responsibility Technology and Innovation Alliance, 2025). The recycling level breakdown based on **WEEE types** are also obtained from (Extended Producer Responsibility Technology and Innovation Alliance, 2025). The **provincial breakdown** within each WEEE type is informed by (Ministry of Ecology and Environment, 2015a) and (Ministry of Ecology and Environment, 2015b), while the CP provincial breakdown is informed by (Wang, 2024).

In the analysis under designed recycling level, the total volume is obtained from (Wang, 2024). The **WEEE type breakdown** are informed by (Ministry of Ecology and Environment, 2015a) and (Ministry of Ecology and Environment, 2015b). The **provincial breakdown** within each WEEE type is based (Wang, 2024).

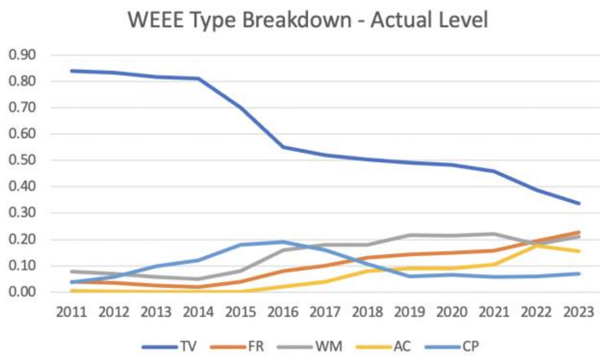
WEEE Type Breakdown - Actual Level
0.90
0.80
0.70
0.60
0.50
0.40
0.30
0.20
0.10
0.00
2011 2012 2013 2014 2015 2016 2017 2018 2019 2020 2021 2022 2023
TV FR WM AC CP

(a)

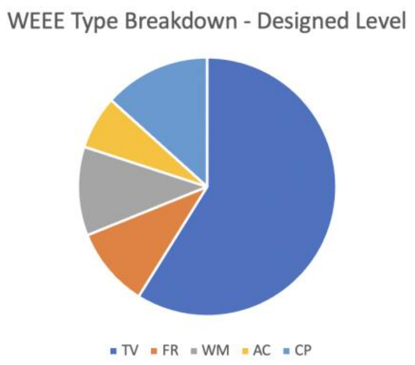
WEEE Type Breakdown - Designed Level
TV FR WM AC CP

(b)

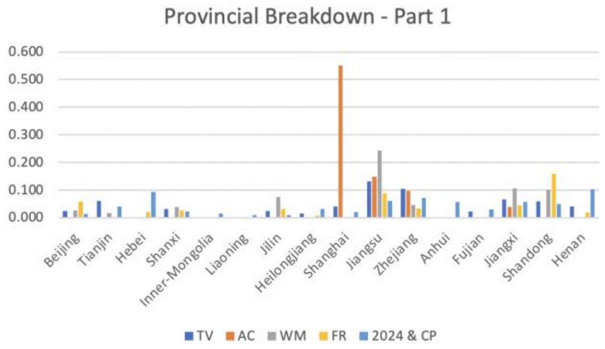
Provincial Breakdown - Part 1
0.600
0.500
0.400
0.300
0.200
0.100
0.000
Beijing Tianjin Hebei Shanxi Inner-Mongolia Liaoning Jilin Heilongjiang Shanghai Jiangsu Zhejiang Anhui Fujian Jiangxi Shandong Henan
TV AC WM FR 2024 & CP

(c)

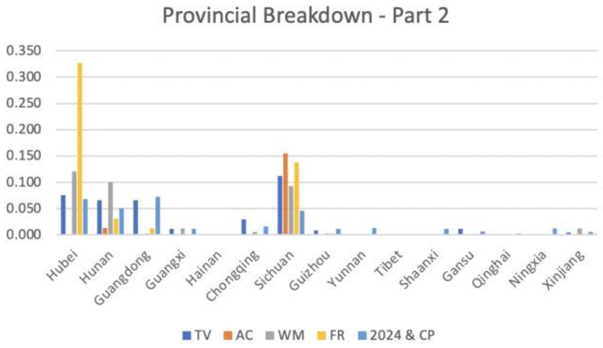
Provincial Breakdown - Part 2
0.350
0.300
0.250
0.200
0.150
0.100
0.050
0.000
Hubei Hunan Guangdong Guangxi Hainan Chongqing Sichuan Guizhou Yunnan Tibet Shaanxi Gansu Qinghai Ningxia Xinjiang
TV AC WM FR 2024 & CP

(d)

Figure S3: Product-specific WEEE generation trajectories for all 14 appliance types from 1978-2050

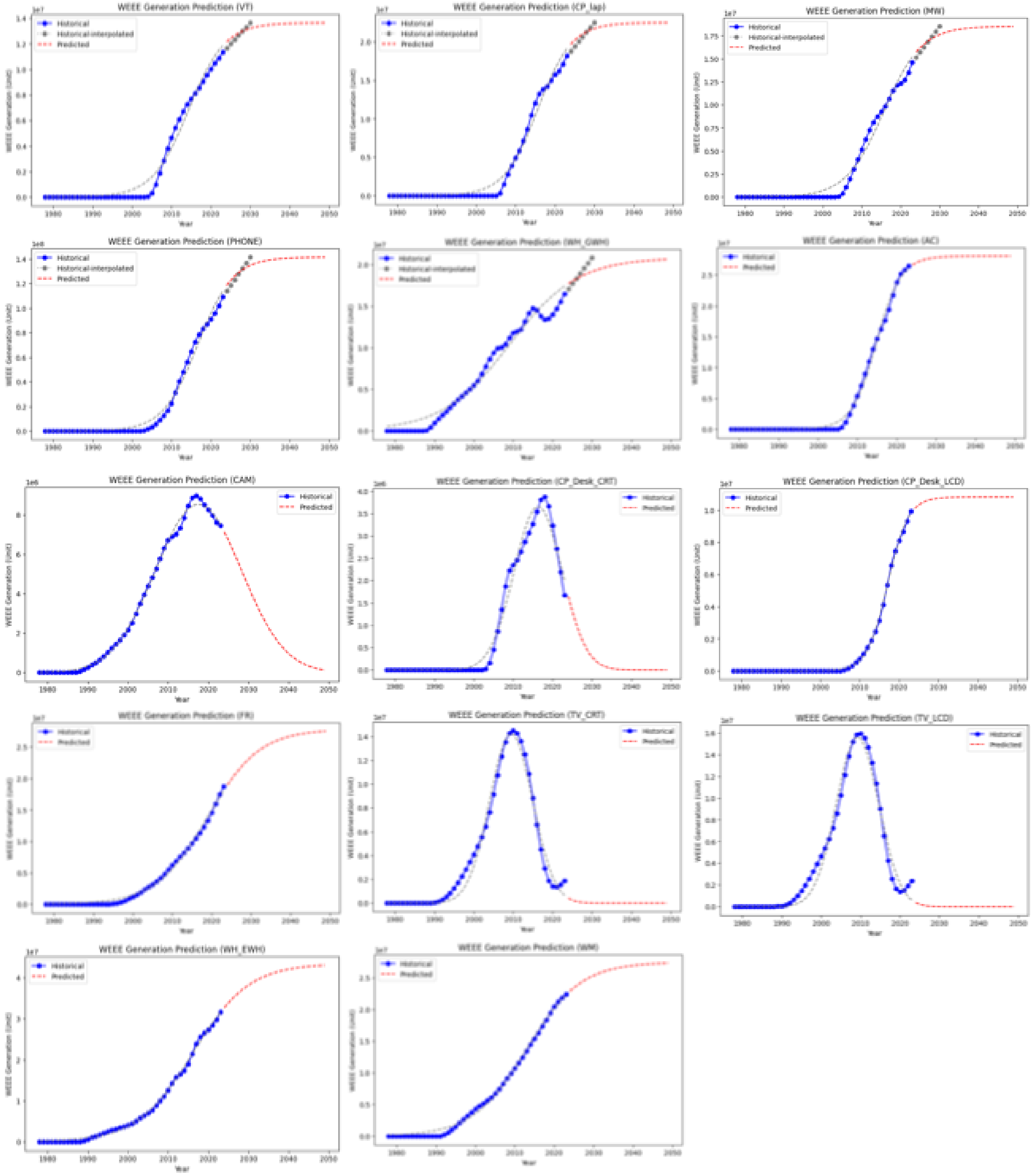

Figure S4: Metal Content Level in Different WEEE Types

<u>Metal Content in WEEEs Without Circuit Boards</u> (unit: KG of the content in per KG of WEEE)

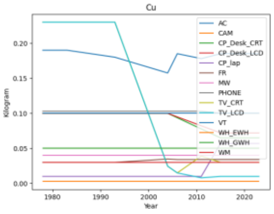

1(a)

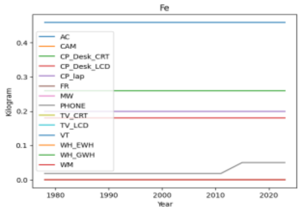

1(b)

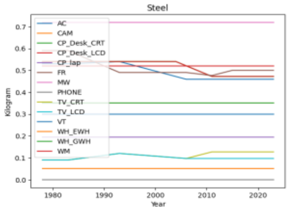

1(c)

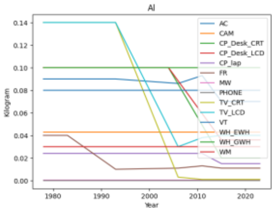

1(d)

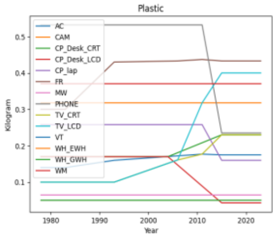

1(e)

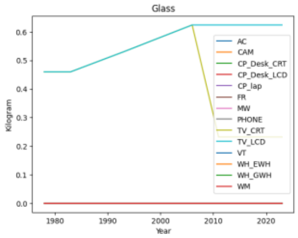

1(f)

Base metals and associated non-metallic materials

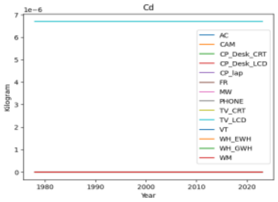

2(a)

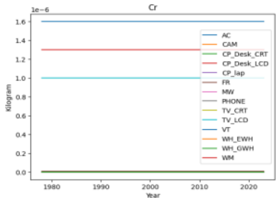

2(b)

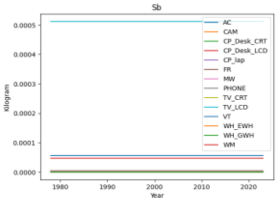

2(c)

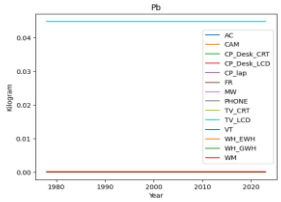

2(d)

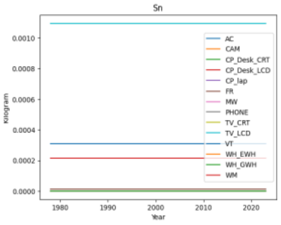

2(e)

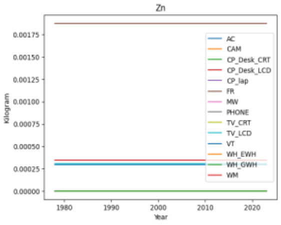

2(f)

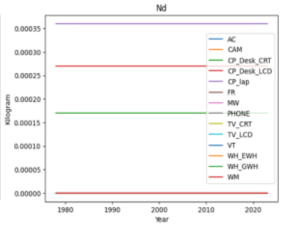

2(g)

Economically valuable metals

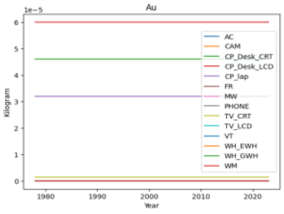

3(a)

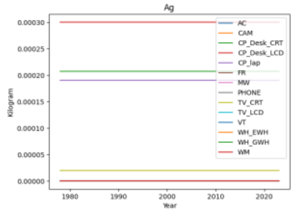

3(b)

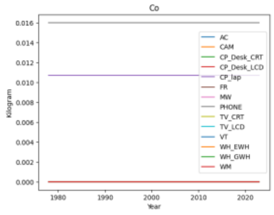

3(c)

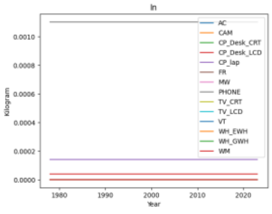

3(d)

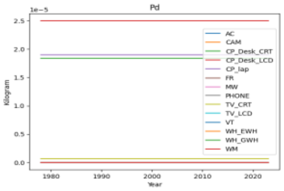

3(e)

Precious metals

*There is no content data of Ba and Ru in WEEE without circuit board.

<u>Metal Content in WEEEs' Circuit Boards Only</u> (unit: KG of the content in per KG of WEEE)

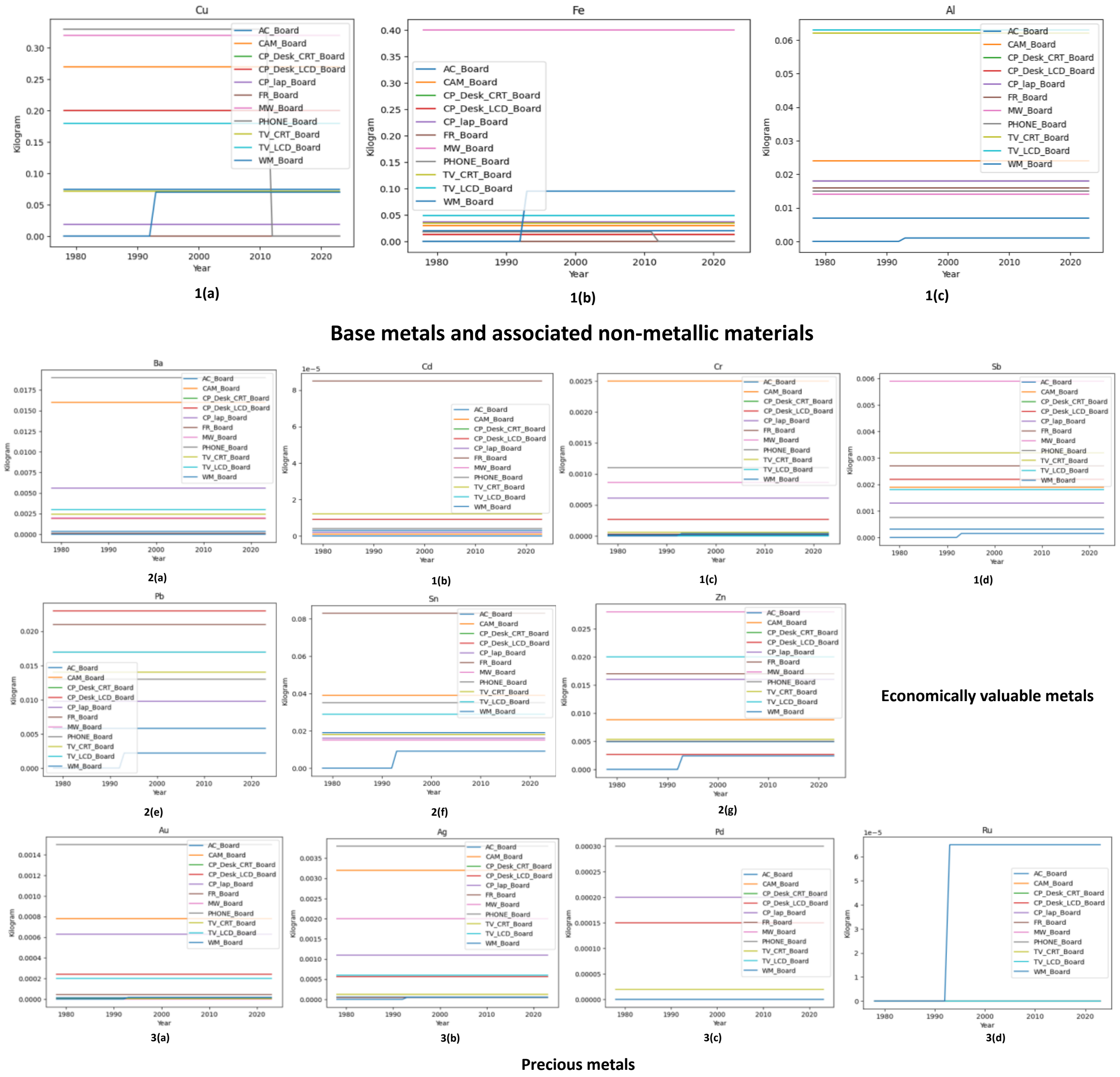

*There is no data on steel, plastic, glass, Nd, Co, and In.

Figure S5: Provincial-level recycling gap for main metals

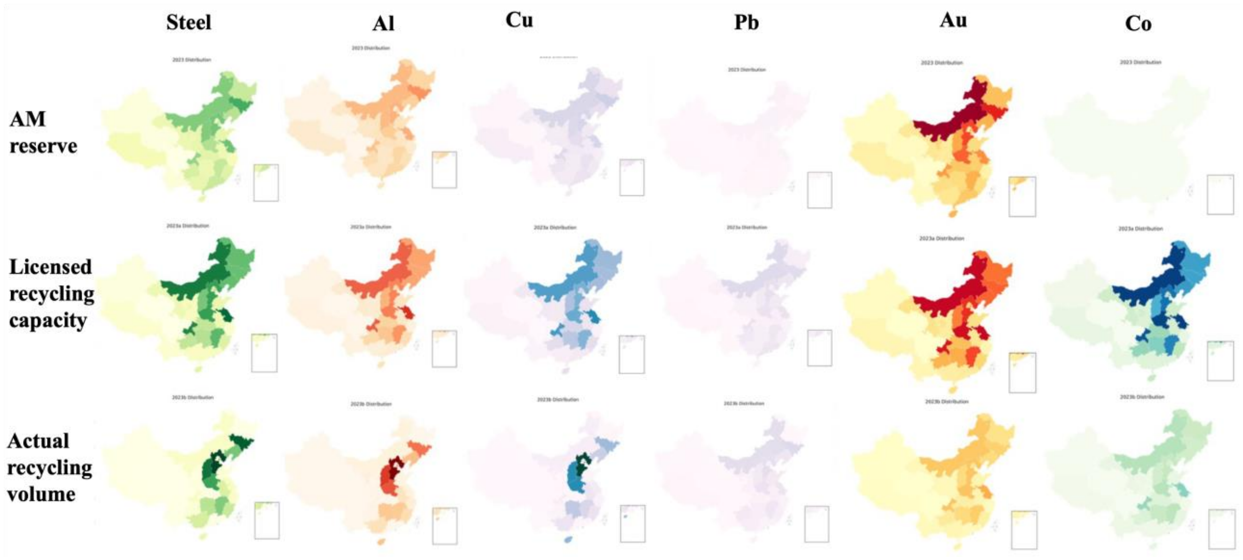

Figure S6: Economic value of metals recovered from household WEEE in China under actual-operation and licensed-capacity scenarios, 2013-2023.

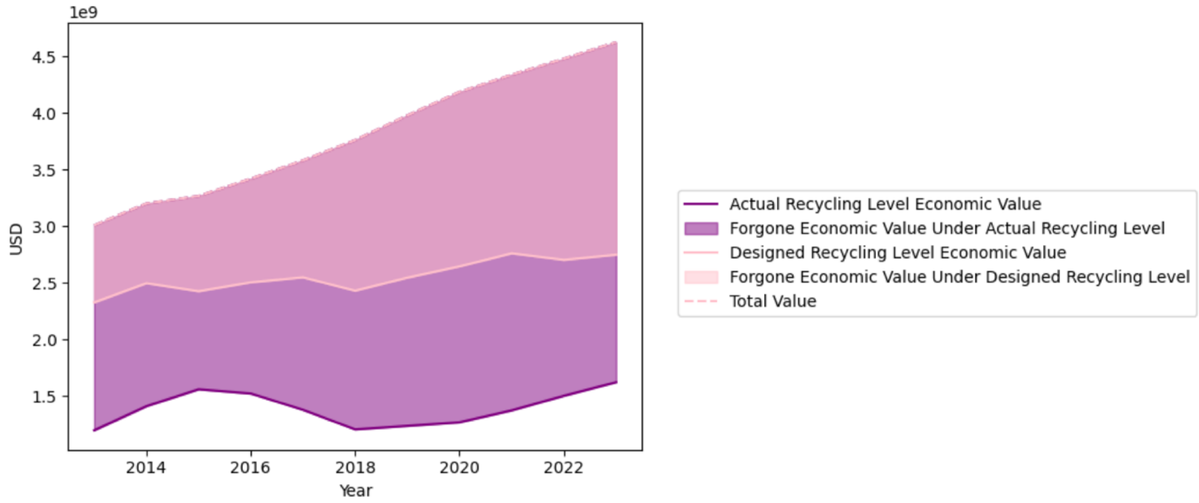

Figure S7: Composition of Recycling Value

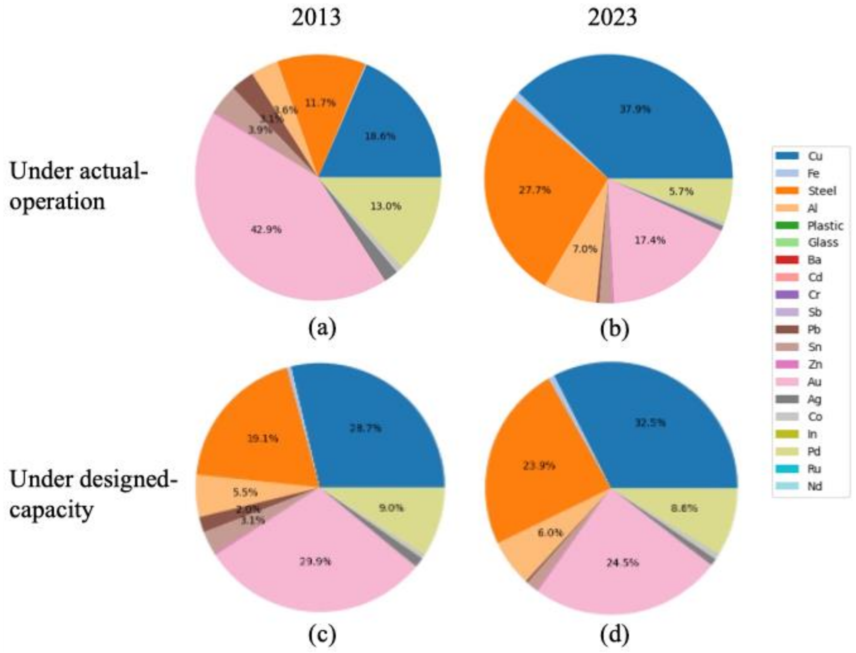

Figure S8: Sensitivity Tests

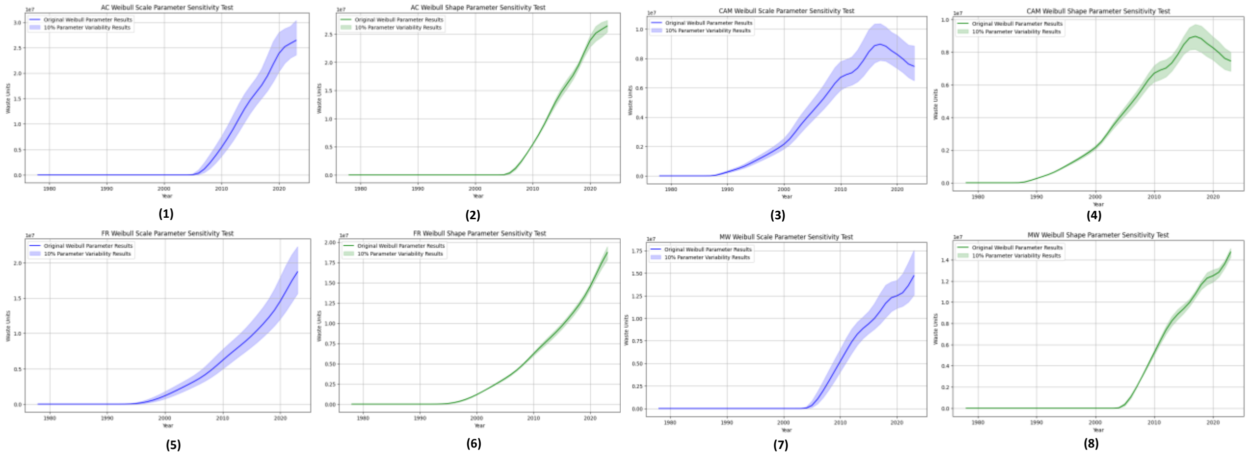

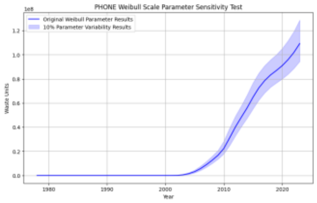

(9)

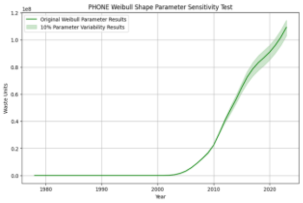

(10)

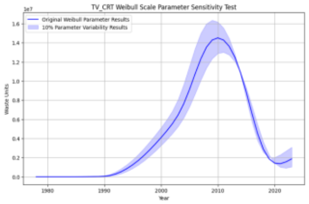

(11)

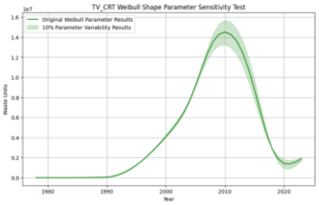

(12)

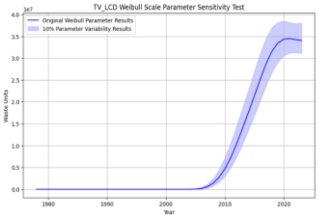

(13)

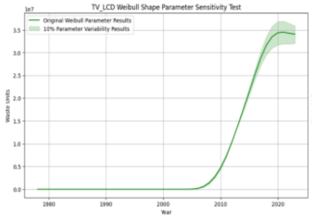

(14)

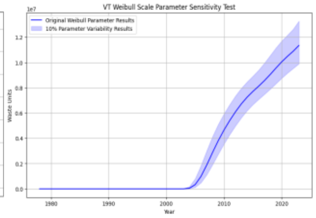

(15)

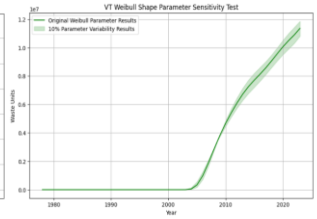

(16)

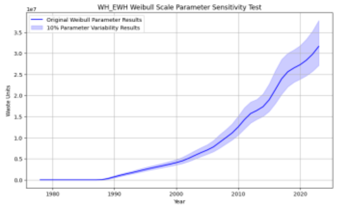

(17)

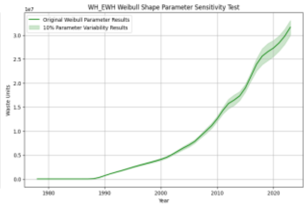

(18)

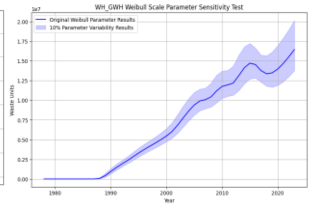

(19)

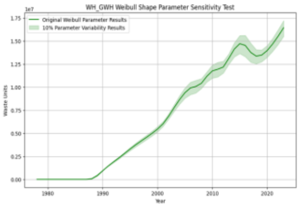

(20)

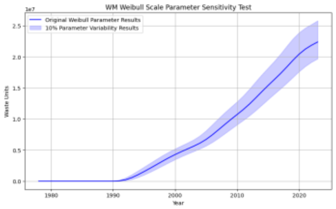

(21)

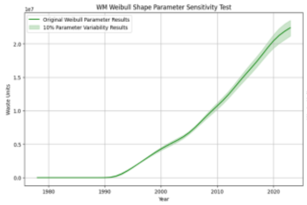

(22)

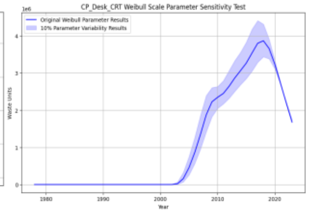

(23)

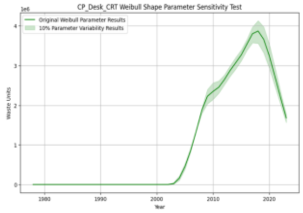

(24)

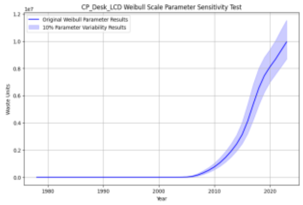

(25)

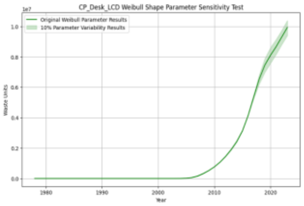

(26)

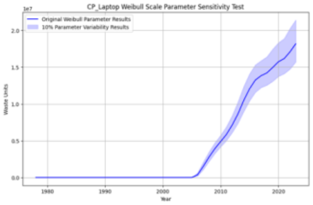

(27)

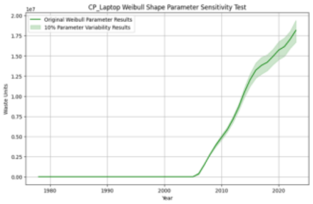

(28)

Figure S9: WEEE Imports

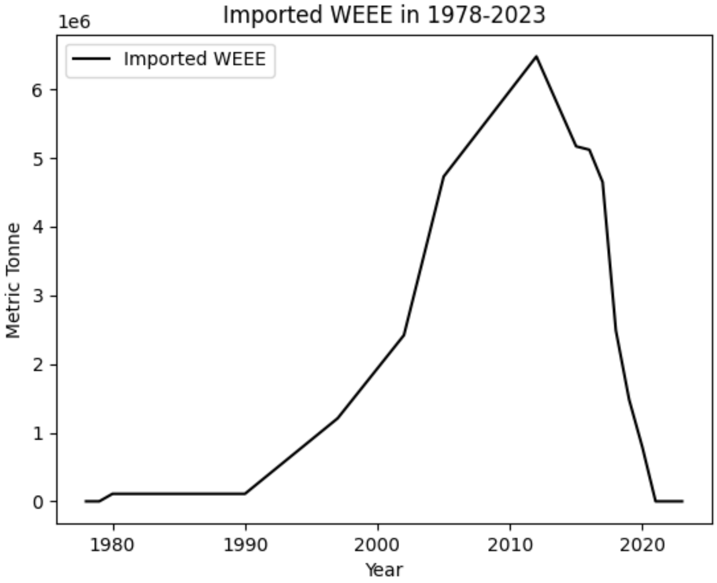

Text S1: Population Statistics

Statistics of population composition and household size for 1998-2023, and the entirety of the statistics on main durable goods owned per 100 households for 1978-2023 are collected directly from the annual China Statistical Yearbooks. Statistics of population composition and household size for 1978-1997 are from the China Population Statistics Yearbook of 1988. Missing data entries are treated with linear interpolation.

Text S2: Results on WEEE Imports

As shown in Figure S6, since 2001, China has banned the importation of 11 types of WEEE including waste television, CRT, computers, and monitors (Xin Hua Net, 2013). However, such illegal activity was not put to an end immediately after 2001 but persisted for over a decade (Zeng et al., 2016). In 2015, a field survey revealed that in Guiyu, a town in Guangdong province, around 1400,000 tonnes of illegally imported WEEE were handled annually (Zeng et al., 2016). This study collected the historic volume of imported WEEE from 1978-2023 based on publicly distributed information from China Customs and the Ministry of Ecology and Environment.

The history of solid waste imports can be traced back to the 1980s when approximately 1 million tonnes of solid waste were imported annually from 1980 to 1990 (Ministry of Ecology and Environment, 2014). Illegal solid waste imports peaked in 2012 at 58.9 million tons and dropped to 47 million tons in 2015 (Ministry of Ecology and Environment, 2014). This corresponds to the peak value of WEEE imports of 6.04 million tons in 2012. Within the solid wastes that were illegally imported, governmental statistics revealed that WEEE takes around 11% (Technology Daily, 2017). Starting from 2021, China fully bans the imports of solid wastes (China Commerce Research Institute, 2020).

Text S2: Composition of Recycling Value

The composition of recycling value also shifts markedly over time (Figure. S7). In 2013, Au was the single largest contributor to the theoretical recycling values, whereas its share declined as the system evolved. Under the actual-operation scenario, Au's share decreased from 42.9% in 2013 to 17.4% in 2023, and under the designed-capacity scenario from 29.9% to 24.5%. This decline is largely driven by changes in the mix of WEEE entering formal recycling: computers and televisions, which contain relatively high Au contents in the circuit boards, account for a smaller share of recycled WEEE over time, while large appliances (e.g., washing machines , refrigerators, air conditioners), dominated by Cu and steel in frames and wiring, become more prevalent.

Correspondingly, the value contribution share of Cu and steel grew significantly. Under actual operation, Cu's contribution to total recycling value rises from 18.6% in 2013 to 37.9% in 2023, and steel from 11.7% to 27.7%. Under the designed-capacity scenario, Cu's share grows from 28.7% to 32.5%, and steel's from 19.1% to 23.9%. Despite these shifts, the top 5 contributing metals to total recycling value remain broadly stable over the decade, indicating that, although the relative importance of specific product categories in formal recycling changes, the dominant materials and manufacturing practices in household EEE have remained relatively consistent.

References

Table S1: In-Use Home Appliance Sub-Type Market Share

Zeng, X., Ali, S. H., Tian, J., & Li, J. (2020). Mapping anthropogenic mineral generation in China and its implications for a circular economy. *Nature Communications*, *11*, 1544. https://doi.org/10.1038/s41467-020-15246-4

Zeng, X., Gong, R., Chen, W.-Q., & Li, J. (2016). Uncovering the Recycling Potential of "New" WEEE in China. *Environmental Science & Technology*, *50*(3), 1347–1358. https://doi.org/10.1021/acs.est.5b05446

Table S2: Weibull Parameters

Zeng, X., Ali, S. H., Tian, J., & Li, J. (2020). Mapping anthropogenic mineral generation in China and its implications for a circular economy. *Nature Communications*, *11*, 1544. https://doi.org/10.1038/s41467-020-15246-4

Forti, V., Baldé, C. P., & Kuehr, R. (2018). *E-waste Statistics: Guidelines on Classifications, Reporting and Indicators, second edition.* . United Nations University, ViE – SCYCLE, Bonn, Germany. . https://collections.unu.edu/eserv/UNU:6477/RZ_EWaste_Guidelines_LoRes.pdf

regulations.gov. *Appendix 8C. LIFETIME DISTRIBUTIONS. EE-2006-STD-0127 COMMENT 41.2*. Retrieved April 14, 2024 from https://downloads.regulations.gov/EERE-2006-STD-0127-0070/attachment_26.pdf

Table S3: WEEE Unit Weight

Zeng, X., Ali, S. H., Tian, J., & Li, J. (2020). Mapping anthropogenic mineral generation in China and its implications for a circular economy. *Nature Communications*, *11*, 1544. https://doi.org/10.1038/s41467-020-15246-4

Bakas, I., Herczeg, M., Vea, E. B., Fråne, A., Youhanan, L., & Baxter, J. (2016). *Critical Metals in Discarded Electronics* Nordic Council of Ministers 2016. https://norden.diva-portal.org/smash/get/diva2:936670/FULLTEXT02.pdf

Kalmykova, Y., Patrício, J., Rosado, L., & EO, B. P. (2015). Out with the old, out with the new – The effect of transitions in TVs and monitors technology on consumption and WEEE generation in Sweden 1996–2014. *Waste Management*, *46*, 511-522. https://doi.org/10.1016/j.wasman.2015.08.034

Forti, V., Baldé, C. P., & Kuehr, R. (2018). *E-waste Statistics: Guidelines on Classifications, Reporting and Indicators, second edition.* . United Nations University, ViE – SCYCLE, Bonn, Germany. . https://collections.unu.edu/eserv/UNU:6477/RZ_EWaste_Guidelines_LoRes.pdf

Balde, C. P., Kuehr, R., Blumenthal, K., Gill, S. F., Kern, M., Micheli, P., Magpantay, E., & Huisman, J. (2015). *E-waste statistics: Guidelines on classifications, reporting and indicators*. United Nations University, IAS - SCYCLE, Bonn, Germany. https://i.unu.edu/media/ias.unu.edu-en/project/2238/E-waste-Guidelines_Partnership_2015.pdf

Table S4: Metal Market Prices

Trading Economics. (2025). *Commodities - live quote price trading data*. Trading Economics. https://tradingeconomics.com/commodities

Figure S5: Metal Content Level in Different WEEE Types

Zeng, X., Ali, S. H., Tian, J., & Li, J. (2020). Mapping anthropogenic mineral generation in China and its implications for a circular economy. *Nature Communications*, *11*, 1544. https://doi.org/10.1038/s41467-020-15246-4

Oguchi, M., Murakami, S., Sakanakura, H., Kida, A., & Kameya, T. (2011). A preliminary categorization of end-of-life electrical and electronic equipment as secondary metal resources. *Waste Management*, *31*(9-10), 2150-2160. https://doi.org/10.1016/j.wasman.2011.05.009

Oguchi, M., Sakanakura, H., & Terazono, A. (2013). Toxic metals in WEEE: Characterization and substance flow analysis in waste treatment processes. *Science of The Total Environment*, *463-464*, 1124-1132. https://doi.org/10.1016/j.scitotenv.2012.07.078

Japan Ministry of the Environment. (2010). *Research Association for Recycling and Proper Treatment of Used Small Home Appliances* Retrieved from https://www.env.go.jp/recycle/recycling/raremetals/conf_ruca.html

Cucchiella, F., D'Adamo, I., Koh, S. C. L., & Rosa, P. (2015). Recycling of WEEEs: An economic assessment of present and future e-waste streams. *Renewable and Sustainable Energy Reviews*, *51*, 263-272. https://doi.org/10.1016/j.rser.2015.06.010

Matsuto, T., Jung, C. H., & Tanaka, N. (2004). Material and heavy metal balance in a recycling facility for home electrical appliances. *24*(5), 425-436. https://doi.org/10.1016/j.wasman.2003.12.002

Kong, Z., & Liu, J. (2018). Stock Material Metabolism of Urban Household Durable Goods in China's Provinces. *International Journal of Ecology*, *7*(3), 153-162. https://doi.org/10.12677/IJE.2018.73017

Source Disclosure for Real Recycling Capacity of Companies

Liu, G., & Wei, H. (2021). *2021 Disposal of Waste Electrical and Electronic Products Industry Research Report*. Ministry of Ecology and Environment, China National Resources Recycling Association, China Association of Electronics Equipments for Technology Development, China Association of Circular Economy Retrieved from http://weee.meescc.cn/test/tzgg/2022-10/files/%E3%80%90%E8%AF%95%E8%AF%BB%E7%89%88%E3%80%91%E5%BA%9F%E5%BC%83%E7%94%B5%E5%99%A8%E7%94%B5%E5%AD%90%E4%BA%A7%E5%93%81%E5%A4%84%E7%90%86%E4%BA%A7%E4%B8%9A%E7%A0%94%E7%A9%B6%E6%8A%A5%E5%91%8A%EF%BC%882021%E5%B9%B4%EF%BC%89.pdf

Extended Producer Responsibility Technology and Innovation Alliance. (2025). *White Papers on WEEE Recycling Industry in China 2012-2024*. Extended Producer Responsibility Technology and Innovation Alliance. Retrieved Jan 1, 2025 from http://www.weee-epr.com/text_23.html

Tian, H. (2012). Current status and prospects of China's waste electrical and electronic products recycling and comprehensive utilization industry. In: China Household Electric Appliances Research Institute.

Yu, K. (2023). 2023 China Waste Electrical and Electronic Products Recycling Industry Development Report. In: China Material Recycling Association.

Designed Recycling Capacity of Companies

Wang, J. (2024). *Summary of the capacity of the 109 government certified waste home appliance dismantling companies in China*. Ecological and Sustainable Development Practices. Retrieved March 15, 2025 from https://mp.weixin.qq.com/s/wKBn5SGIYcXUOCOtux1oUg

Type and Province Breakdown of Recycled WEEE

Ministry of Ecology and Environment. (2015a). *2013 Quarter 1 and 2 Statistics on WEEE Successful Recycling Rate and Stipend Claim.* . Ministry of Ecology and Environment, Retrieved from https://www.mee.gov.cn/ywgz/gtfwyhxpgl/fqdqdzcpcjclqksh/201604/P020160424387307376079.pdf

Ministry of Ecology and Environment. (2015b). *2013 Quarter 3 and 4 Statistics on WEEE Successful Recycling Rate and Stipend Claim.* . Ministry of Ecology and Environment, Retrieved from https://www.mee.gov.cn/ywgz/gtfwyhxpgl/fqdqdzcpcjclqksh/201604/P020160424387327920509.pdf

Wang, J. (2024). *Summary of the capacity of the 109 government certified waste home appliance dismantling companies in China*. Ecological and Sustainable Development Practices. Retrieved March 15, 2025 from https://mp.weixin.qq.com/s/wKBn5SGIYcXUOCOtux1oUg
Liu, G., & Wei, H. (2021). *2021 Disposal of Waste Electrical and Electronic Products Industry Research Report*. Ministry of Ecology and Environment, China National Resources Recycling Association, China Association of Electronics Equipments for Technology Development, China Association of Circular Economy Retrieved from http://weee.meescc.cn/test/tzgg/2022-10/files/%E3%80%90%E8%AF%95%E8%AF%BB%E7%89%88%E3%80%91%E5%BA%9F%E5%BC%83%E7%94%B5%E5%99%A8%E7%94%B5%E5%AD%90%E4%BA%A7%E5%93%81%E5%A4%84%E7%90%86%E4%BA%A7%E4%B8%9A%E7%A0%94%E7%A9%B6%E6%8A%A5%E5%91%8A%EF%BC%882021%E5%B9%B4%EF%BC%89.pdf
Extended Producer Responsibility Technology and Innovation Alliance. (2025). *White Papers on WEEE Recycling Industry in China 2012-2024*. Extended Producer Responsibility Technology and Innovation Alliance. Retrieved Jan 1, 2025 from http://www.weee-epr.com/text_23.html
Tian, H. (2012). Current status and prospects of China's waste electrical and electronic products recycling and comprehensive utilization industry. In: China Household Electric Appliances Research Institute.
Yu, K. (2023). 2023 China Waste Electrical and Electronic Products Recycling Industry Development Report. In: China Material Recycling Association.

Figure S6: WEEE Type and Provincial Breakdown in Recycling

Extended Producer Responsibility Technology and Innovation Alliance. (2025). *White Papers on WEEE Recycling Industry in China 2012-2024*. Extended Producer Responsibility Technology and Innovation Alliance. Retrieved Jan 1, 2025 from http://www.weee-epr.com/text_23.html
Ministry of Ecology and Environment. (2015a). *2013 Quarter 1 and 2 Statistics on WEEE Successful Recycling Rate and Stipend Claim.* . Ministry of Ecology and Environment, Retrieved from https://www.mee.gov.cn/ywgz/gtfwyhxpgl/fqdqdzcpcjclqksh/201604/P020160424387307376079.pdf
Ministry of Ecology and Environment. (2015b). *2013 Quarter 3 and 4 Statistics on WEEE Successful Recycling Rate and Stipend Claim.* . Ministry of Ecology and Environment, Retrieved from https://www.mee.gov.cn/ywgz/gtfwyhxpgl/fqdqdzcpcjclqksh/201604/P020160424387327920509.pdf
Wang, J. (2024). *Summary of the capacity of the 109 government certified waste home appliance dismantling companies in China*. Ecological and Sustainable Development Practices. Retrieved March 15, 2025 from https://mp.weixin.qq.com/s/wKBn5SGIYcXUOCOtux1oUg

Section S9: Results on WEEE Imports

China Commerce Research Institute. (2020). Imported waste reform has achieved significant results: solid waste imports decreased by 41% year-on-year. . *China Environmental News*. https://res.cenews.com.cn/hjw/news.html?aid=134683
Ministry of Ecology and Environment. (2014). *Environmental Protection Control Standards for Imported Solid Waste as Raw Materials*. Retrieved from https://www.mee.gov.cn/gkml/hbb/bgth/201409/W020140912376837561288.pdf
Ministry of Ecology and Environment. (2020). *Transcript of the Ministry of Ecology and Environment's November Regular Press Conference*. Ministry of Ecology and Environment Retrieved from https://www.mee.gov.cn/xxgk2018/xxgk/xxgk15/202011/t20201130_810582_wh.html#:~:text=一是固体废物进口,、51.4%25 和 71%25%E3%80%82
Technology Daily. (2017). Environmental Hazards in Countries that Import Large Volume of Wastes: There is no free "lunch" *Xin Hua Net*. http://www.xinhuanet.com/politics/2017-02/14/c_1120464202.htm
Xin Hua Net. (2013). 70% of electronic waste returns to China. Who "opens the door" to foreign waste? *Xin Hua Net*. http://www.cciced.net/xwzx/hfyw/201308/t20130801_83212.html
Zeng, X., Gong, R., Chen, W.-Q., & Li, J. (2016). Uncovering the Recycling Potential of "New" WEEE in China. *Environmental Science & Technology*, *50*(3), 1347–1358. https://doi.org/10.1021/acs.est.5b05446
